# Um Estudo de Randomização Mendeliana de Duas Amostras sobre Poluição do Ar/Tabagismo e Parada Cardíaca

**DOI:** 10.36660/abc.20250127

**Published:** 2026-03-26

**Authors:** Xuelin Tian, Yu Wang, Qingzhao Tan, Liying Liu

**Affiliations:** 1 Zhongshan Hospital Xiamen University Xiamen China Zhongshan Hospital Xiamen University, Xiamen – China; 2 Xiamen University Department of Emergency Xiamen China Xiamen University - Department of Emergency, Xiamen – China

**Keywords:** Poluição do Ar, Tabagismo, Parada Cardíaca, Análise da Randomização Mendeliana

## Abstract

**Fundamento:**

Estudos observacionais sugeriram uma ligação entre poluição do ar/tabagismo e parada cardíaca (PC); no entanto, o papel causal da poluição do ar/tabagismo na PC permanece incerto.

**Objetivos:**

Este estudo teve como objetivo elucidar a associação causal entre poluição do ar/tabagismo e PC usando uma abordagem de randomização mendeliana (RM) de duas amostras.

**Métodos:**

Este estudo utilizou dados resumidos de estudos de associação genômica ampla (GWAS). A análise primária empregou o método de ponderação pela variância inversa (IVW), complementado pelos métodos MR-Egger, mediana ponderada e moda ponderada. Para a análise de sensibilidade, os métodos MR-Egger e MR-PRESSO foram utilizados para lidar com a pleiotropia horizontal. O teste Q de Cochran foi usado para avaliar a heterogeneidade, e a análise de *leave-one-out* foi aplicada para identificar a influência de outliers. Um valor de p bicaudal < 0,05 foi considerado estatisticamente significativo para as estimativas causais.

**Resultados:**

O método IVW mostrou uma associação significativa entre o status de tabagismo e a PC (OR = 2,182, IC de 95%:1,137– 4.188, p = 0,019). Não foram encontradas associações causais significativas para nenhuma das métricas de poluição do ar ou outras características relacionadas ao tabagismo. Os resultados dos métodos MR Egger e mediana ponderada revelaram uma associação causal insignificante entre poluição do ar/tabagismo e PC. Pleiotropia horizontal ou outliers não foram detectados pelos testes de sensibilidade.

**Conclusões:**

Os resultados deste estudo de randomização mendeliana indicam que o tabagismo tem um efeito causal na incidência do desenvolvimento de PC. No entanto, os resultados nulos para a poluição do ar devem ser interpretados com cautela, visto que o poder estatístico para detectar efeitos modestos foi limitado. Mais estudos são necessários para verificar os resultados deste estudo.

## Graphical abstract

**Figura Central:**
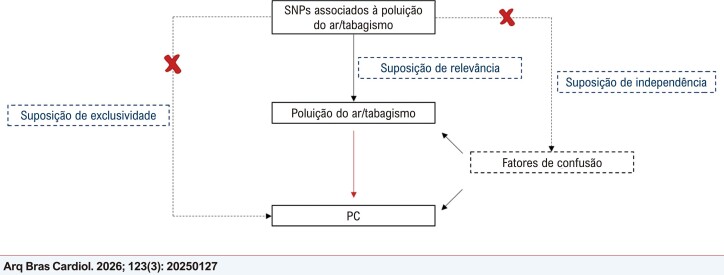
Um Estudo de Randomização Mendeliana de Duas Amostras sobre Poluição do Ar/Tabagismo e Parada Cardíaca. Descrição do desenho do estudo. Um fluxograma ilustra como a análise de Randomização Mendeliana foi conduzida neste estudo. PC: parada cardíaca.

## Introdução

A parada cardíaca (PC) significa a falência da atividade mecânica cardíaca, associada à ausência de evidências clínicas observáveis de circulação sistêmica.^1^ Globalmente, a PC é responsável por cerca de 15% de todas as fatalidades e representa metade, ou 50%, de todas as mortes relacionadas a problemas cardíacos.^2^ As diferenças fisiopatológicas subjacente e nos sistemas de atendimento exigem o exame da epidemiologia por meio de duas categorias distintas: parada cardiorrespiratória intra-hospitalar e parada cardiorrespiratória extra-hospitalar (PCREH).^1^ A PCREH, definida como a cessação da atividade mecânica cardíaca que ocorre fora do ambiente hospitalar, é a condição mais comum e crítica, caracterizada por uma baixa taxa de sobrevida. Fatores de estilo de vida, como tabagismo, níveis de atividade física e dieta, também têm sido associados à PC.^3^ Além disso, a crescente preocupação levou ao reconhecimento de que os fatores ambientais desempenham um papel crucial no início das PCREHs; consequentemente, a associação entre a exposição aguda a poluentes atmosféricos e a incidência de PCREHs se tornou um ponto central das pesquisas recentes.

Em um estudo prospectivo que examinou a associação entre o tabagismo e a cessação do tabagismo no risco de morte súbita cardíaca (MSC) em 101.018 mulheres, em comparação com as não fumantes, as fumantes atuais apresentaram um risco 2,44 vezes maior de PC (intervalo de confiança de 95% IC: 1,80 - 3,31), após ajustes para fatores de risco coronariano. Em análises multifatoriais, tanto a quantidade diária de cigarros fumados (P para tendência <0,0001) quanto a duração do tabagismo (P para tendência <0,0001) demonstraram correlações lineares com o aumento do risco de PC entre as fumantes atuais, correspondendo a um aumento de 8% no risco de PC (razão de risco (RR) = 1,08; IC 95% = 1,05–1,12; P < 0,0001).^4^ Um estudo anterior documentou que a cessação do tabagismo pode levar a uma diminuição substancial do risco de PC.^5^ Em um estudo multicêntrico de caso-controle realizado em 17 hospitais na Coreia, os resultados mostraram que o tabagismo atual estava associado à incidência de PC (razão de chances ajustada (OR) = 1,39, IC 95%: 1,08–1,79).^6^ Além disso, pesquisas epidemiológicas têm revelado consistentemente um risco elevado de doenças cardiovasculares e mortalidade associado à presença de material particulado (MP) na poluição do ar ambiente.^7 , 8^ Em particular, estudos anteriores investigaram a correlação entre os níveis de poluição do ar e a incidência de PCREH. O risco de PCREH foi correlacionado com elevações de curto prazo na exposição a MP, especificamente partículas finas com diâmetro aerodinâmico inferior a 2,5 micrômetros (MP2,5) ou MP ultrafinas, e, por vezes, partículas mais grossas com diâmetros inferiores a 10 micrômetros (MP10), juntamente com poluentes gasosos como o ozônio (O_3_), que podem ter origem em diversas fontes, incluindo atividades industriais e emissões veiculares.^9^

No entanto, os resultados de estudos observacionais são suscetíveis à influência de fatores de confusão, principalmente relacionados às características basais e ao desenho do estudo. A Randomização Mendeliana (RM) é uma estratégia sofisticada de variável instrumental (VI) que utiliza polimorfismos de nucleotídeo único (SNPs) como VIs naturais inerentes, facilitando a análise das relações causais entre características fenotípicas.^10^ Esta metodologia mitiga inerentemente os vieses decorrentes de variáveis de confusão e contorna a questão da causalidade reversa, aumentando assim a credibilidade das relações causais inferidas. Este estudo teve como objetivo investigar a relação causal entre a poluição do ar/tabagismo e o risco de PC. Os resultados deste estudo podem auxiliar na elucidação dos mecanismos genéticos e das vias biológicas implicadas na PC.

## Métodos

### Desenho do estudo

O presente estudo de RM foi conduzido sob três pressupostos fundamentais: (1) Relevância: A variante genética está significativamente associada à exposição em investigação; (2) Independência: A variante genética é independente de quaisquer fatores de confusão que possam influenciar a relação entre a exposição e o desfecho; e (3) Restrição de Exclusão: A variante genética impacta o desfecho unicamente por meio de sua associação com a exposição, não exercendo nenhum efeito independente direto.^11^ Este estudo empregou uma análise RM de duas amostras para avaliar a associação causal entre poluição do ar/tabagismo e o risco de PC ( Figura Central ).

### Fonte de dados

Neste estudo, a PC foi considerada o desfecho, e a poluição do ar e o tabagismo foram considerados a exposição. Os dados resumidos sobre PC foram obtidos do conjunto de dados FinnGen. O tamanho da amostra foi de 118.055. Os dados sobre tabagismo foram obtidos do projeto IEU Open GWAS (https://gwas.mrcieu.ac.uk/) e os dados sobre poluição do ar foram obtidos da *Medical Research Council-Integrative Epidemiology Unit* (MRC-IEU).^12^ O modelo de regressão de uso do solo (LUR) do projeto ESCAPE foi utilizado para estimar as concentrações de poluentes atmosféricos nas proximidades das residências, que foram então integradas aos dados genômicos do sequenciamento sanguíneo disponível no conjunto de dados da coorte do UK Biobank (UKB).^13 , 14^ Informações detalhadas são fornecidas na Tabela S1 . Todos os dados são de domínio público, e a aprovação ética e o consentimento informado foram obtidos nos estudos originais.

### Seleção de variáveis instrumentais (VIs)

Para atender às premissas mencionadas, o banco de dados GWAS foi consultado para a seleção de SNPs. Posteriormente, um limiar de significância de P < 5 × 10^-6^ foi utilizado para selecionar SNPs como potenciais variáveis instrumentais (VIs). Esse limiar ligeiramente menos rigoroso, em comparação com o nível de significância genômica estrito (P < 5 × 10^-8^), é uma estratégia comumente empregada em estudos de RM para aumentar o número de instrumentos disponíveis, aprimorando assim o poder estatístico da análise, especialmente para exposições com um número limitado de resultados significativos em todo o genoma.^15^ O critério de inclusão para a frequência do alelo minoritário (MAF) das variantes em investigação foi definido em 0,01.^10^ Todos os SNPs identificados foram submetidos a agrupamento para evitar problemas de desequilíbrio de ligação, utilizando um parâmetro de janela de agrupamento rigoroso definido com um limite de R^2^ de 0,001 e um kb de 10.000.^9^ Quando as variáveis instrumentais estavam ausentes no conjunto de dados de resultados, foram buscadas e utilizadas substitutas com alto desequilíbrio de ligação (DL), com um R^2^ superior a 0,8.^16^ Para avaliar a força do instrumento e lidar com o potencial viés de instrumento fraco, a estatística F foi calculada para cada SNP dentro do conjunto de variáveis instrumentais. Esse cálculo seguiu a fórmula: $F=\left[R^{2}*(N-2)\right]\text{ / (1 - }R^{2})$ , em que R^2^ representa a proporção da variância na exposição explicada pelo SNP e N representa o tamanho da amostra.^17^ Para garantir a robustez do instrumento e evitar viés de instrumento fraco, apenas os SNPs que apresentaram um valor F > 10 foram mantidos.^17^

### Randomização Mendeliana

A análise RM de duas amostras foi conduzida utilizando o software R, versão 4.3.3, empregando os pacotes TwoSampleMR e MR-PRESSO. A análise primária de RM foi executada utilizando o método da ponderação pela variância inversa (IVW), partindo do pressuposto de que não há efeitos pleiotrópicos médios. O método IVW é considerado a abordagem mais eficaz para estimar a relação causal entre poluição do ar/tabagismo e PC, utilizando razões de chances (ORs) juntamente com seus respectivos IC de 95% para quantificar a associação. Para avaliar a robustez dos nossos achados, métodos analíticos adicionais, incluindo regressão MR-Egger, mediana ponderada e moda ponderada, foram empregados como análises complementares. A abordagem MR-Egger incorpora um termo de intercepto, permitindo a obtenção de estimativas não viesadas dos efeitos causais mesmo na presença de pleiotropia horizontal.^18^ A abordagem da mediana ponderada se baseia na premissa de que pelo menos metade das variáveis instrumentais estima com precisão a relação causal entre a exposição e o desfecho.^19^ A estratégia de modo ponderado identifica o efeito causal concentrando-se em um subconjunto de SNPs que exibem a maior densidade de efeitos, o que é conseguido agrupando os SNPs de acordo com a similaridade nos impactos causais inferidos.^20^ Foram realizados ajustes para testes múltiplos utilizando o procedimento de taxa de descoberta falsa (FDR) de Benjamini-Hochberg, levando em consideração o número efetivo de características independentes.^21^ Um gráfico de dispersão foi usado para representar a associação entre poluição do ar/tabagismo e PC. Um gráfico de floresta foi empregado para mostrar a análise de MR e o IC de 95% para cada SNP.

### Análise de sensibilidade

Foram realizadas análises de sensibilidade para verificar a confiabilidade das estimativas de MR. Na análise IVW, o teste I^2^ e o teste Q de Cochran foram empregados para avaliar a heterogeneidade entre os efeitos dos SNPs associados aos desfechos de PC, com um valor de p superior a 0,05 indicando ausência de heterogeneidade estatisticamente significativa.^22^ A regressão MR-Egger é empregada para detectar pleiotropia potencial e avaliar seu impacto na estimativa de risco, sendo o teste de intercepto fundamental. Um valor p > 0,05 sugere ausência de pleiotropia significativa.^23^ Um gráfico de funil foi utilizado para inspecionar visualmente e ilustrar a presença de pleiotropia horizontal. Além disso, o método MR-PRESSO ( *MR Pleiotropy Residual Sum and Outlier* ) foi empregado para avaliar sistematicamente a pleiotropia horizontal e identificar e, posteriormente, remover quaisquer SNPs discrepantes que pudessem distorcer a inferência causal.^24^ Além disso, após a exclusão de variáveis instrumentais (VIs) discrepantes, examinamos se havia uma diferença estatisticamente significativa nos resultados em comparação com os resultados anteriores à sua remoção. Uma análise de validação cruzada *leave-one-out* (LOO) foi realizada para determinar a influência de cada SNP na relação causal geral, garantindo assim a robustez de nossas descobertas.

### Poder estatístico

O poder estatístico da nossa análise de MR foi avaliado usando a ferramenta online “mRnd” (https://shiny.cnsgenomics.com/mRnd/), que é especificamente projetada para cálculos de poder em análises de variáveis instrumentais. Essas calculadoras estimam o poder para detectar um efeito de determinada magnitude para um desfecho contínuo, com base em vários parâmetros-chave: (1) a proporção da variância na exposição explicada pela VI (R^2^), (2) o verdadeiro tamanho do efeito causal, (3) o tamanho da amostra do GWAS do desfecho, (4) o número de instrumentos genéticos utilizados e (5) o nível de significância pré-especificado (α).^25^

## Resultados

### Seleção de VIs

Este estudo identificou 1138 variáveis instrumentais (VIs) associadas à poluição do ar e ao tabagismo. Ao calcular as estatísticas F para essas VIs, a estatística F média foi de 29,26. É importante ressaltar que a estatística F mínima para qualquer SNP incluído foi de 20,34, valor substancialmente superior ao limiar convencional de 10, indicando que nossas análises provavelmente não foram afetadas por viés de instrumento fraco ( Tabela S2 ). Na análise RM com PC como desfecho, 56 SNPs não foram pareados nos dados resumidos e 19 SNPs não tinham SNPs substitutos apropriados ( Tabela S3 ).

### Randomização Mendeliana

**Figura 1:**
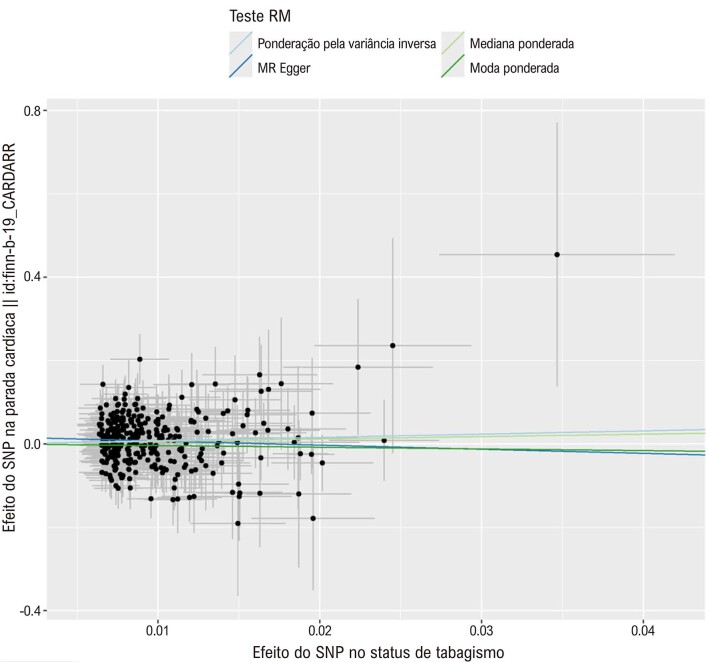
– Gráficos de dispersão da associação entre o status de tabagismo e a parada cardíaca. As inclinações representam as associações causais, com cada linha representando uma metodologia diferente.

Os resultados do IVW mostraram uma associação significativa entre o status de tabagismo e a PC (OR = 2,182, IC de 95%:1,137– 4.188, p = 0,019). As Figuras 1 e 2 apresentam os diagramas de dispersão e os diagramas de floresta do efeito do status de tabagismo na PC. Além disso, os resultados do IVW não mostraram evidências de uma relação causal entre várias métricas de poluição do ar (incluindo MP2,5, absorbância de MP2,5, MP10 e dióxido de nitrogênio) ou outras características relacionadas ao tabagismo (tabagismo prévio, idade de início do tabagismo e nunca ter fumado) e o risco de PC. Os resultados detalhados dessas análises são apresentados na Tabela 1 . O gráfico de dispersão para os resultados negativos é mostrado nas Figuras S1-S2 , e o gráfico de floresta para os resultados negativos é mostrado nas Figuras S3-S4 .

**Figura 2:**
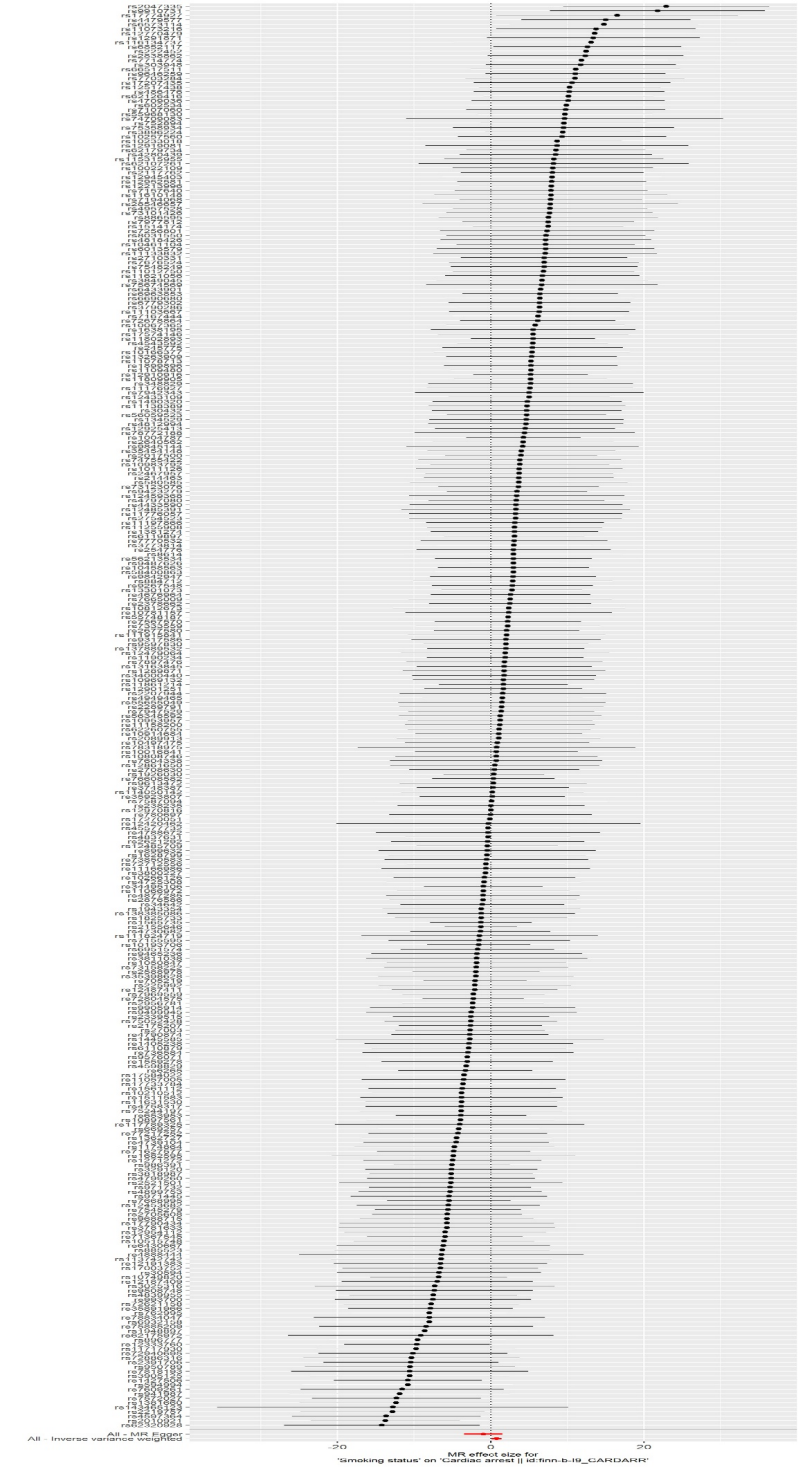
Gráficos de floresta da associação entre o status de tabagismo e a parada cardíaca. A estimativa de RM derivada dos métodos MR-Egger e IVW foi apresentada para comparação.

**Tabela 1:** Resultados da relação causal entre poluição do ar/tabagismo e parada cardíaca

Exposição	Resultado	Significado do SNP	N. SNPs	Métodos	OR (IC 95%)	p
Status de tabagismo	Parada cardíaca	5*10-6	317	MR Egger	0,374 ( 0,031, 4,501 )	0,439
Status de tabagismo	Parada cardíaca		317	Mediana ponderada	1,793 ( 0,669, 4,804 )	0,245
Status de tabagismo	Parada cardíaca		317	Ponderação pela variância inversa	2,182 ( 1,137, 4,188 )	0,019
Status de tabagismo	Parada cardíaca		317	Modo ponderado	0,673 ( 0,039, 11,63 )	0,786
Idade de iniciação ao tabagismo	Parada cardíaca		50	MR Egger	2,091 ( 0,243, 17,968)	0,505
Idade de iniciação ao tabagismo	Parada cardíaca		50	Mediana ponderada	1,424 ( 0,439, 4,622 )	0,556
Idade de iniciação ao tabagismo	Parada cardíaca		50	Ponderação pela variância inversa	1,208 ( 0,517, 2,823 )	0,663
Idade de iniciação ao tabagismo	Parada cardíaca		50	Modo ponderado	4,125 ( 0,355, 47,936 )	0,263
Poluição atmosférica por partículas finas (MP2,5)	Parada cardíaca		55	MR Egger	0,383 ( 0,081, 1,81 )	0,231
Poluição atmosférica por partículas finas (MP2,5)	Parada cardíaca		55	Mediana ponderada	0,386 ( 0,078, 1,909 )	0,243
Poluição atmosférica por partículas finas (MP2,5)	Parada cardíaca		55	Ponderação pela variância inversa	0,683 ( 0,269, 1,736 )	0,424
Poluição atmosférica por partículas finas (MP2,5)	Parada cardíaca		55	Moda ponderada	0,435 ( 0,092, 2,051 )	0,298
Absorbância da poluição atmosférica por partículas finas (MP2,5)	Parada cardíaca		56	MR Egger	0,717 ( 0,193, 2,658 )	0,621
Absorbância da poluição atmosférica por partículas finas (MP2,5)	Parada cardíaca		56	Mediana ponderada	0,819 ( 0,2, 3,358 )	0,781
Absorbância da poluição atmosférica por partículas finas (MP2,5)	Parada cardíaca		56	Ponderação pela variância inversa	0,658 ( 0,275, 1,577 )	0,348
Absorbância da poluição atmosférica por partículas finas (MP2,5)	Parada cardíaca		56	Moda ponderada	0,547 ( 0,157, 1,906 )	0,348
Poluição atmosférica por partículas finas (MP10)	Parada cardíaca		28	MR Egger	0,228 ( 0,004, 11,78 )	0,469
Poluição atmosférica por partículas finas (MP10)	Parada cardíaca		28	Mediana ponderada	0,73 ( 0,071, 7,477 )	0,791
Poluição atmosférica por partículas finas (MP10)	Parada cardíaca		28	Ponderação pela variância inversa	1,234 ( 0,187, 8,123 )	0,827
Poluição atmosférica por partículas finas (MP10)	Parada cardíaca		28	Moda ponderada	0,53 ( 0,012, 24,257 )	0,747
Histórico de tabagismo	Parada cardíaca		241	MR Egger	0,967 ( 0,214, 4,373 )	0,965
Histórico de tabagismo	Parada cardíaca		241	Mediana ponderada	0,747 ( 0,414, 1,348 )	0,333
Histórico de tabagismo	Parada cardíaca		241	Ponderação pela variância inversa	1,007 ( 0,681, 1,489 )	0,972
Histórico de tabagismo	Parada cardíaca		241	Moda ponderada	0,508 ( 0,116, 2,221 )	0,369
Poluição atmosférica por dióxido de nitrogênio	Parada cardíaca		98	MR Egger	0,877 ( 0,286, 2,694 )	0,819
Poluição atmosférica por dióxido de nitrogênio	Parada cardíaca		98	Mediana ponderada	0,996 ( 0,302, 3,289 )	0,995
Poluição atmosférica por dióxido de nitrogênio	Parada cardíaca		98	Ponderação pela variância inversa	0,582 ( 0,281, 1,202 )	0,143
Poluição atmosférica por dióxido de nitrogênio	Parada cardíaca		98	Moda ponderada	1,473 ( 0,458, 4,741 )	0,518
Tabagismo: Nunca fumou	Parada cardíaca		226	MR Egger	6,496 (0,221, 191,07)	0,279
Tabagismo: Nunca fumou	Parada cardíaca		226	Mediana ponderada	1,433 ( 0,39, 5,267 )	0,588
Tabagismo: Nunca fumou	Parada cardíaca		226	Ponderação pela variância inversa	1,075 ( 0,435, 2,654 )	0,876
Tabagismo: Nunca fumou	Parada cardíaca		226	Moda ponderada	1,996 (0,054, 73,131)	0,707
Poluição atmosférica por partículas de 2,5 a 10 μm	Parada cardíaca		6	MR Egger	0,798 ( 0,356, 1,79 )	0,614
Poluição atmosférica por partículas de 2,5 a 10 μm	Parada cardíaca		6	Mediana ponderada	1,198 ( 0,698, 2,057 )	0,513
Poluição atmosférica por partículas de 2,5 a 10 μm	Parada cardíaca		6	Ponderação pela variância inversa	1,162 ( 0,766, 1,764 )	0,481
Poluição atmosférica por partículas de 2,5 a 10 μm	Parada cardíaca		6	Moda ponderada	1,222 ( 0,647, 2,306 )	0,564

### Análises de sensibilidade

**Figura 3:**
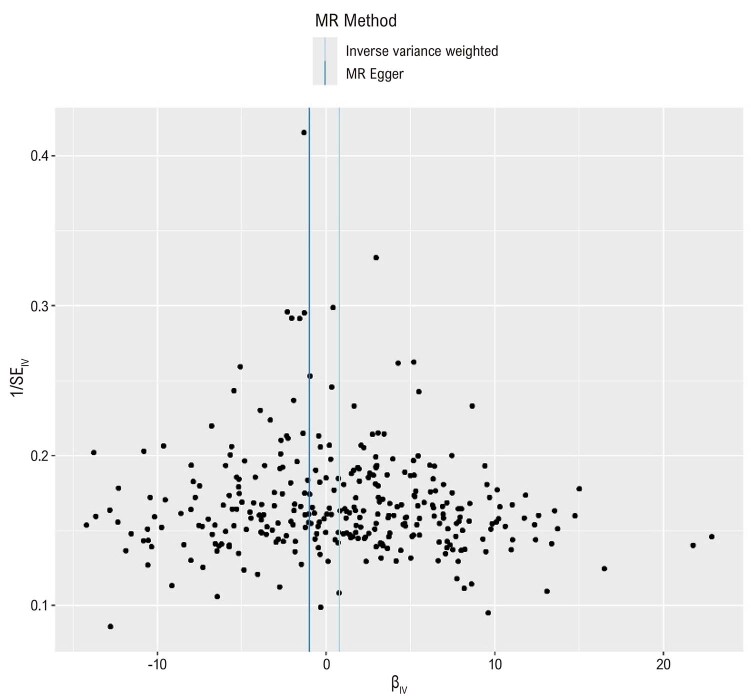
– Gráficos de funil da associação entre o status de tabagismo e a PC. Cada ponto representa uma variante genética, plotada de acordo com o tamanho do efeito estimado e o erro padrão. O gráfico ajuda a identificar assimetrias, que podem indicar potenciais vieses, como pleiotropia ou viés de publicação.

A análise de regressão MR-Egger indicou ausência de viés devido à pleiotropia horizontal ( Tabela S4 ). Foi detectada heterogeneidade na análise que relaciona a poluição atmosférica por partículas (MP2,5) à PC, enquanto nenhuma heterogeneidade foi observada nas demais análises ( Tabela S4 ). As Figuras 3 e 4 apresentam os gráficos de funil e os gráficos *leave-one-out* do efeito do status de tabagismo na PC. As Figuras S5-S6 representam os gráficos de funil dos resultados negativos, e as Figuras S7-S8 mostram os gráficos *leave-one-out* dos resultados negativos. De acordo com a análise MR-PRESSO, um SNP discrepante (rs1537371) foi identificado na associação entre MP2,5 e PC; no entanto, mesmo após a exclusão deste SNP, nenhuma associação estatisticamente significativa persistiu. Nenhum outro valor discrepante foi detectado nas demais análises. ( Tabela S5 ).

**Figura 4:**
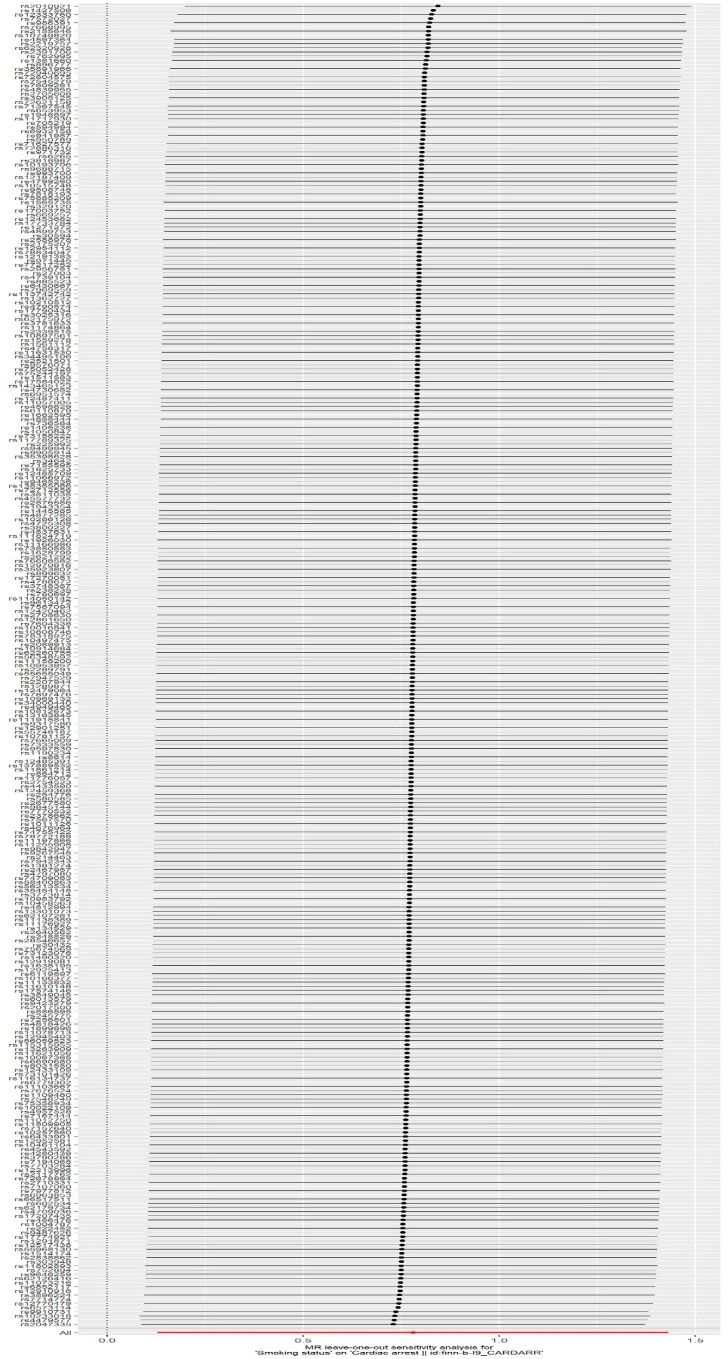
– Gráficos de *leave-one-out* da associação entre o tabagismo e a PC. Cada ponto indica o tamanho do efeito estimado quando uma variante genética é removida da análise. As linhas horizontais representam os intervalos de confiança de 95%, enquanto a linha vertical mostra a estimativa do efeito agrupado geral.

### Poder estatístico

Os cálculos de poder estatístico para nossas análises de RM são apresentados na Tabela S6 . Os resultados indicam um poder estatístico geralmente baixo (<80%) para detectar efeitos causais na maioria das associações exposição-desfecho investigadas.

## Discussão

Até onde sabemos, esta é a primeira análise RM a examinar se a poluição do ar/tabagismo tem um papel causal no desenvolvimento de PC. Os resultados deste estudo revelaram uma conexão causal entre uma predisposição genética ao tabagismo e a PC. Além disso, nosso estudo identificou que a poluição do ar não está causalmente relacionada à PC.

Diversas investigações avaliaram a associação entre o risco de MSC e a adesão a práticas de estilo de vida saudáveis. Dentre esses fatores, o tabagismo se destaca como o atributo de estilo de vida mais frequentemente examinado em relação à MSC.^5 , 26^ Em um estudo de coorte prospectivo com mulheres sem doença arterial coronariana no começo, foi descoberta uma forte relação dose-resposta entre o tabagismo e o risco de doença arterial coronariana. Além disso, se demonstrou que a cessação do tabagismo levou a uma redução substancial e, por fim, à eliminação do risco elevado de doença arterial coronariana.^4^ Embora os resultados estivessem de acordo com o presente estudo, a população incluída foi limitada a mulheres. Em um estudo caso-controle realizado por Park et al., o não tabagismo demonstrou ser um fator de proteção contra o risco de PC (OR = 0,36, IC 95%: 0,27–0,47) após ajuste para idade, sexo, área de residência, comorbidades cardiovasculares e hábitos de vida saudáveis.^27^ Além disso, em uma metanálise recente que incluiu 12 estudos prospectivos, o risco relativo (RR) agrupado foi estimado em 3,06 (IC 95%: 2,46 – 3,82) para indivíduos que fumam atualmente, em comparação com aqueles que nunca fumaram.^28^ Aproximadamente 80% das PCs podem ser atribuídas a taquiarritmias ventriculares, e uma maior vulnerabilidade tanto a episódios iniciais quanto a recorrências dessas arritmias, bem como à fibrilação ventricular entre fumantes, pode ser atribuída a distúrbios nos índices de dispersão do tempo de repolarização ventricular.^29 - 31^ A nicotina, um componente chave dos cigarros, demonstrou em estudos com animais provocar um espectro de distúrbios do ritmo cardíaco, incluindo pausas sinusais transitórias, bradicardia, taquicardia sinusal, fibrilação atrial, bloqueio sinoatrial, bloqueio atrioventricular e taquiarritmias ventriculares, o que reforça seu papel na fisiopatologia do risco arritmogênico relacionado ao tabagismo.^32^ Os resultados deste estudo reforçaram a importância de políticas e estratégias populacionais para o abandono do tabagismo na redução da incidência de mortes súbitas cardíacas.

Em estudos observacionais anteriores, as concentrações de poluentes atmosféricos, incluindo MP2,5, MP10, NO_2_ e O_3_, foram identificadas como fatores de risco para PC.^26^ Kang et al. realizaram uma análise para avaliar os efeitos da exposição elevada a MP10 e MP2,5 na ocorrência de PCREH em um contexto populacional. Seus resultados revelaram uma correlação significativa entre níveis mais altos de exposição a MP2,5 e um risco aumentado de PCREH.^33^ Um estudo realizado em Estocolmo, na Suécia, revelou uma notável relação dose-resposta, associando um maior risco de PCREH a níveis elevados de O_3_, enquanto nenhuma associação semelhante foi observada para MP2,5 ou NO_2_.^34^ Por outro lado, um estudo separado sobre PCREH realizado na Lombardia, Itália, empregando metodologias distintas, demonstrou que as concentrações de todos os poluentes estudados estavam significativamente elevadas em dias com alta incidência de PCREH, com exceção do O_3_.^35^

Uma recente revisão sistemática e metanálise abrangendo 15 estudos que investigaram a relação entre PCREH e a exposição a diversos poluentes atmosféricos, com foco principal nas concentrações de MP10, MP2,5, NO_2_ e O_3_, revelou uma ligação significativa entre o aumento do risco de PC e níveis elevados de MP2,5, MP10 e O_3_ no ambiente.^36^ Da mesma forma, outro estudo de Sielski et al. também relatou a influência de MP2,5 e MP10 nos parâmetros de PCREH.^37^ A disparidade em alguns dos resultados dos estudos mencionados pode ser atribuída às diferentes metodologias de estudo, características dos participantes, métodos de medição dos poluentes, misturas de exposições e graus de suscetibilidade individual. É importante notar que os resultados de nossas análises de RM revelaram uma relação causal insignificante entre poluentes atmosféricos, incluindo MP10, MP2,5, NO_2_ e MP de 2,5 a 10 μm. Uma consideração primordial na interpretação dessas associações nulas é o poder estatístico de nossa análise. Como elucidam nossos cálculos de poder ( Tabela S6 ), o estudo, em geral, não teve poder suficiente para detectar efeitos causais de pequena a moderada intensidade para as métricas de poluição do ar. Portanto, a ausência de uma associação estatisticamente significativa não deve ser interpretada como evidência definitiva de ausência de efeito causal, mas sim como um reflexo das limitações do presente estudo em detectar tal relação. Os mecanismos subjacentes aos achados insignificantes em relação à relação causal entre poluição do ar e PC podem ser variações na magnitude ou no caráter da resposta inflamatória, decorrentes de diferenças na composição química das partículas e na duração ou intensidade das exposições. Algumas pessoas provavelmente são mais vulneráveis, com evidências sugerindo que aquelas com riscos cardiovasculares subjacentes e síndrome metabólica podem apresentar associações mais fortes.^38^ Por outro lado, medicamentos anti-inflamatórios, como as estatinas, podem neutralizar os efeitos das partículas ambientais.^30^ A magnitude e a consistência dessas alterações variam entre os diferentes biomarcadores e populações de pacientes estudados.^8^ É importante notar que a RM estima o efeito causal de níveis de exposição ao longo da vida, geneticamente previstos. Em contraste, muitos estudos observacionais associaram a PC a picos agudos e de curto prazo na poluição do ar. É plausível que a exposição crônica a níveis mais baixos (representada pela genética) não tenha um efeito causal detectável, enquanto a exposição aguda a níveis elevados atue como um gatilho para eventos cardíacos em indivíduos suscetíveis. Nosso estudo não foi projetado para capturar tais efeitos de curto prazo. Além disso, a relação entre poluição do ar e PC pode ser não linear, com o risco aumentando significativamente apenas acima de um determinado limiar de exposição não capturado pelos instrumentos genéticos. Embora a RM seja robusta contra muitos fatores de confusão, não podemos descartar completamente mediadores não mensurados ou vias de pleiotropia horizontal. Por exemplo, o status socioeconômico é um fator de confusão complexo associado tanto aos níveis de poluição do ar residencial quanto à saúde cardiovascular, e seus efeitos podem não ser totalmente capturados.

Nosso estudo apresenta vários pontos fortes. Ele contribui com informações valiosas para a compreensão da relação causal entre poluição do ar/tabagismo e PC, oferecendo uma base empírica para pesquisas futuras nessa área. Além disso, ao questionar a noção de que o tabagismo possa ser um fator de risco confiável para PC, nossas descobertas trazem implicações importantes. No entanto, reconhecemos algumas limitações deste estudo. Primeiramente, nossa análise se restringiu a coortes de ascendência europeia, o que limita a generalização de nossos resultados. A arquitetura genética, como frequências alélicas e padrões de desequilíbrio de ligação, pode diferir entre populações. Além disso, as interações gene-ambiente podem variar; por exemplo, o impacto da predisposição genética ao tabagismo pode ser modificado por diferentes normas sociais ou níveis de exposição a outros fatores de risco em populações não europeias. Portanto, futuros estudos de RM em diversos grupos ancestrais, como aqueles de ascendência africana ou asiática, são cruciais para validar nossas descobertas e avaliar suas implicações para a saúde pública global. Em segundo lugar, embora não possamos excluir definitivamente a sobreposição de participantes entre os conjuntos de dados para exposição e desfecho, quantificar essa sobreposição com precisão representa um desafio metodológico. Apesar disso, a elevada robustez de nossas variáveis instrumentais, evidenciada por estatísticas F > 10, atenua significativamente o potencial viés decorrente de qualquer sobreposição, estando em consonância com os critérios estabelecidos.^17^ Em terceiro lugar, uma limitação significativa é o poder estatístico insuficiente para várias de nossas análises, particularmente para as exposições à poluição do ar. Isso limita nossa capacidade de tirar conclusões firmes a partir dos resultados nulos e destaca que a ausência de evidência não é evidência de ausência. Essa questão ressalta a necessidade de estudos futuros utilizando conjuntos de dados resumidos de GWAS maiores, o que proporcionaria maior poder para detectar potenciais efeitos causais de pequena ou moderada intensidade. Dadas essas limitações, a interpretação cautelosa dos resultados deste estudo é justificada, ressaltando a necessidade de estudos futuros que abordem essas limitações e expandam o escopo da investigação para populações mais amplas e com um conjunto mais abrangente de SNPs para aumentar a precisão e a generalização dos resultados.

## Conclusões

Os resultados desta análise RM de duas amostras fornecem evidências de uma ligação causal geneticamente inferida entre o tabagismo e parada cardíaca. Estratégias específicas para a cessação do tabagismo são necessárias para reduzir a ocorrência de parada cardíaca. Para consolidar os resultados do presente estudo, análises futuras devem empregar conjuntos de dados resumidos de estudos de associação genômica ampla (GWAS) maiores, baseados em múltiplas populações e um repertório expandido de instrumentos genéticos.

## Material suplementar

Figura(s) Suplementar(es)

Tabela(s) suplementar(es)

## References

[1] Gill R, Pitcher M, Ruland S (2021). Handb Clin Neurol.

[2] Albert CM, Stevenson WG, Jameson JL, Fauci AS, Kasper DL, Hauser SL, Longo DL, Loscalzo J (2018). Harrison's Principles of Internal Medicine.

[3] Chiuve SE, Fung TT, Rexrode KM, Spiegelman D, Manson JE, Stampfer MJ (2011). Adherence to a Low-Risk, Healthy Lifestyle and Risk of Sudden Cardiac Death among Women. JAMA.

[4] Sandhu RK, Jimenez MC, Chiuve SE, Fitzgerald KC, Kenfield SA, Tedrow UB (2012). Smoking, Smoking Cessation, and Risk of Sudden Cardiac Death in Women. Circ Arrhythm Electrophysiol.

[5] Goldenberg I, Jonas M, Tenenbaum A, Boyko V, Matetzky S, Shotan A (2003). Current Smoking, Smoking Cessation, and the Risk of Sudden Cardiac Death in Patients with Coronary Artery Disease. Arch Intern Med.

[6] Jung E, Park JH, Ro YS, Ryu HH, Cha KC, Do Shin S (2023). Family History, Socioeconomic Factors, Comorbidities, Health Behaviors, and the Risk of Sudden Cardiac Arrest. Sci Rep.

[7] Brook RD, Franklin B, Cascio W, Hong Y, Howard G, Lipsett M (2004). Air Pollution and Cardiovascular Disease: A Statement for Healthcare Professionals from the Expert Panel on Population and Prevention Science of the American Heart Association. Circulation.

[8] Brook RD, Rajagopalan S, Pope CA, Brook JR, Bhatnagar A, Diez-Roux AV (2010). Particulate Matter air Pollution and Cardiovascular Disease: An Update to the Scientific Statement from the American Heart Association. Circulation.

[9] Abecasis GR, Altshuler D, Auton A, Brooks LD, Durbin RM, Gibbs RA (2010). A Map of Human Genome Variation from Population-Scale Sequencing. Nature.

[10] Long Y, Tang L, Zhou Y, Zhao S, Zhu H (2023). Causal Relationship between Gut Microbiota and Cancers: A Two-Sample Mendelian Randomisation Study. BMC Med.

[11] Burgess S, Smith GD, Davies NM, Dudbridge F, Gill D, Glymour MM (2023). Guidelines for Performing Mendelian Randomization Investigations: Update for Summer 2023. Wellcome Open Res.

[12] Battram T, Yousefi P, Crawford G, Prince C, Sheikhalil Babei M, Sharp G (2022). The EWAS Catalog: A Database of Epigenome-Wide Association Studies. Wellcome Open Res.

[13] Montagne D, Hoek G, Nieuwenhuijsen M, Lanki T, Pennanen A, Portella M (2014). The Association of LUR Modeled PM2.5 Elemental Composition with Personal Exposure. Sci Total Environ.

[14] Sudlow C, Gallacher J, Allen N, Beral V, Burton P, Danesh J (2015). UK Biobank: An Open Access Resource for Identifying the Causes of a Wide Range of Complex Diseases of Middle and Old Age. PLoS Med.

[15] Ference BA, Majeed F, Penumetcha R, Flack JM, Brook RD (2015). Effect of Naturally Random Allocation to Lower Low-Density Lipoprotein Cholesterol on the Risk of Coronary Heart Disease Mediated by Polymorphisms in NPC1L1, HMGCR, or Both: A 2 × 2 Factorial Mendelian Randomization Study. J Am Coll Cardiol.

[16] Deng MG, Liu F, Liang Y, Wang K, Nie JQ, Liu J (2023). Association between Frailty and Depression: A Bidirectional Mendelian Randomization Study. Sci Adv.

[17] Burgess S, Thompson SG, CRP CHD Genetics Collaboration (2011). Avoiding Bias from Weak Instruments in Mendelian Randomization Studies. Int J Epidemiol.

[18] Bowden J, Smith GD, Burgess S (2015). Mendelian Randomization with Invalid Instruments: Effect Estimation and Bias Detection Through Egger Regression. Int J Epidemiol.

[19] Bowden J, Smith GD, Haycock PC, Burgess S (2016). Consistent Estimation in Mendelian Randomization with Some Invalid Instruments Using a Weighted Median Estimator. Genet Epidemiol.

[20] Hartwig FP, Smith GD, Bowden J (2017). Robust Inference in Summary data Mendelian Randomization Via the Zero Modal Pleiotropy Assumption. Int J Epidemiol.

[21] Benjamini Y, Hochberg Y (2018). Controlling the False Discovery Rate: A Practical and Powerful Approach to Multiple Testing. J R Statist Soc B.

[22] Greco MF D, Minelli C, Sheehan NA, Thompson JR (2015). Detecting Pleiotropy in Mendelian Randomisation Studies with Summary Data and a Continuous Outcome. Stat Med.

[23] Burgess S, Thompson SG (2017). Interpreting Findings from Mendelian Randomization Using the MR-Egger Method. Eur J Epidemiol.

[24] Verbanck M, Chen CY, Neale B, Do R (2018). Detection of Widespread Horizontal Pleiotropy in Causal Relationships Inferred from Mendelian Randomization between Complex Traits and Diseases. Nat Genet.

[25] Brion MJ, Shakhbazov K, Visscher PM (2013). Calculating Statistical Power in Mendelian Randomization Studies. Int J Epidemiol.

[26] Adabag AS, Luepker RV, Roger VL, Gersh BJ (2010). Sudden Cardiac Death: Epidemiology and Risk Factors. Nat Rev Cardiol.

[27] Park JH, Cha KC, Ro YS, Song KJ, Shin SD, Jung WJ (2022). Healthy Lifestyle Factors, Cardiovascular Comorbidities, and the Risk of Sudden cardiac Arrest: A Case-Control Study in Korea. Resuscitation.

[28] Aune D, Schlesinger S, Norat T, Riboli E (2018). Tobacco Smoking and the Risk of Sudden Cardiac Death: A Systematic Review and Meta-Analysis of Prospective Studies. Eur J Epidemiol.

[29] Ghaeeart PJ, De Buyzere ML, Taeymans YM, Gillebert TC, Henriques JP, Backer G (2006). Risk Factors for Primary Ventricular Fibrillation during Acute Myocardial Infarction: A Systematic Review and Meta-Analysis. Eur Heart J.

[30] Goldenberg I, Moss AJ, McNitt S, Zareba W, Daubert JP, Hall WJ (2006). Cigarette Smoking and the Risk of Supraventricular and Ventricular Tachyarrhythmias in High-Risk Cardiac Patients with Implantable Cardioverter Defibrillators. J Cardiovasc Electrophysiol.

[31] Plank B, Kutyifa V, Moss AJ, Huang DT, Ruwald AC, McNitt S (2014). Smoking is Associated with an Increased Risk of First and Recurrent Ventricular Tachyarrhythmias in Ischemic and Nonischemic Patients with Mild Heart Failure: A MADIT-CRT Substudy. Heart Rhythm.

[32] D'Alessandro A, Boeckelmann I, Hammwöhner M, Goette A (2012). Nicotine, Cigarette Smoking and Cardiac Arrhythmia: An Overview. Eur J Prev Cardiol.

[33] Kang SH, Heo J, Oh Y, Kim J, Lim WH, Cho Y (2016). Ambient Air Pollution and Out-of-Hospital Cardiac Arrest. Int J Cardiol.

[34] Raza A, Bellander T, Bero-Bedada G, Dahlquist M, Hollenberg J, Jonsson M (2014). Short-Term Effects of Air Pollution on Out-of-Hospital Cardiac Arrest in Stockholm. Eur Heart J.

[35] Gentile FR, Primi R, Baldi E, Compagnoni S, Mare C, Contri E (2021). Out-of-hospital Cardiac Arrest and Ambient Air Pollution: A Dose-Effect Relationship and an Association with OHCA Incidence. PLoS One.

[36] Zhao R, Chen S, Wang W, Huang J, Wang K, Liu L (2017). The Impact of Short-Term Exposure to Air Pollutants on the Onset of Out-of-Hospital Cardiac Arrest: A Systematic Review and Meta-Analysis. Int J Cardiol.

[37] Sielski J, Kaziród-Wolski K, Józwiak MA, Józwiak M (2021). The Influence of Air Pollution by PM2.5, PM10 and Associated Heavy Metals on the Parameters of Out-of-Hospital Cardiac Arrest. Sci Total Environ.

[38] Mehra R (2007). Global public health problem of sudden cardiac death. J Electrocardiol.

