# Supplementary material for: Um Estudo de Randomização Mendeliana de Duas Amostras sobre Poluição do Ar/Tabagismo e Parada Cardíaca
Source: Arq Bras Cardiol. 2026 Mar 26;123(3):20250127. [Article in Portuguese] doi: 10.36660/abc.20250127 (PMC13128207; doi:10.36660/abc.20250127)
Supplement: Figura(s) Suplementar(es) [file 0066-782x-abc-123-3-20250127-suppl1.pdf]

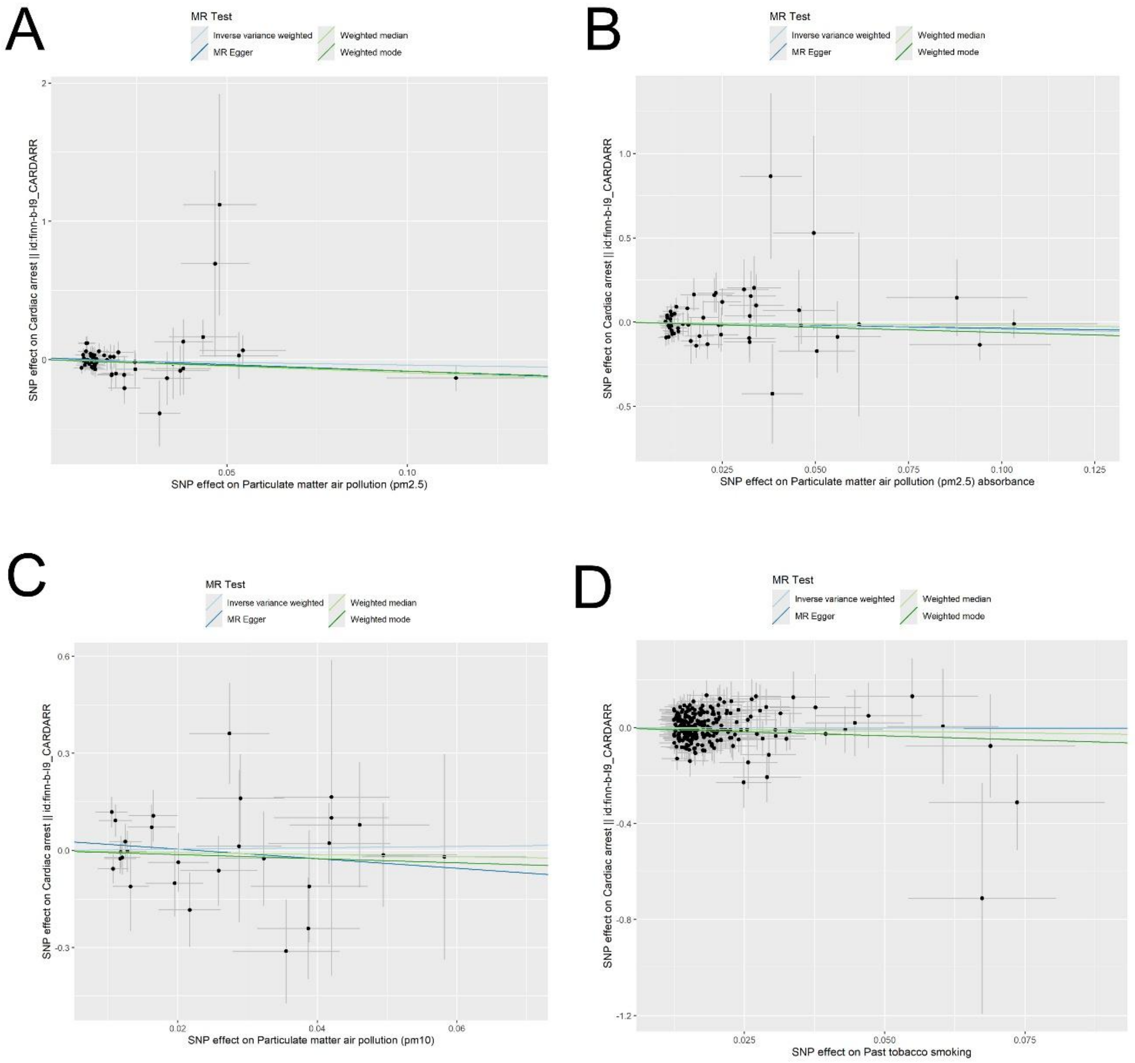

Figure S1. Scatter plots of the association between particulate matter air pollution (PM2.5) (A), particulate matter air pollution (PM2.5) absorbance (B), particulate matter air pollution (PM10)(C), past tobacco smoking(D) and CA.

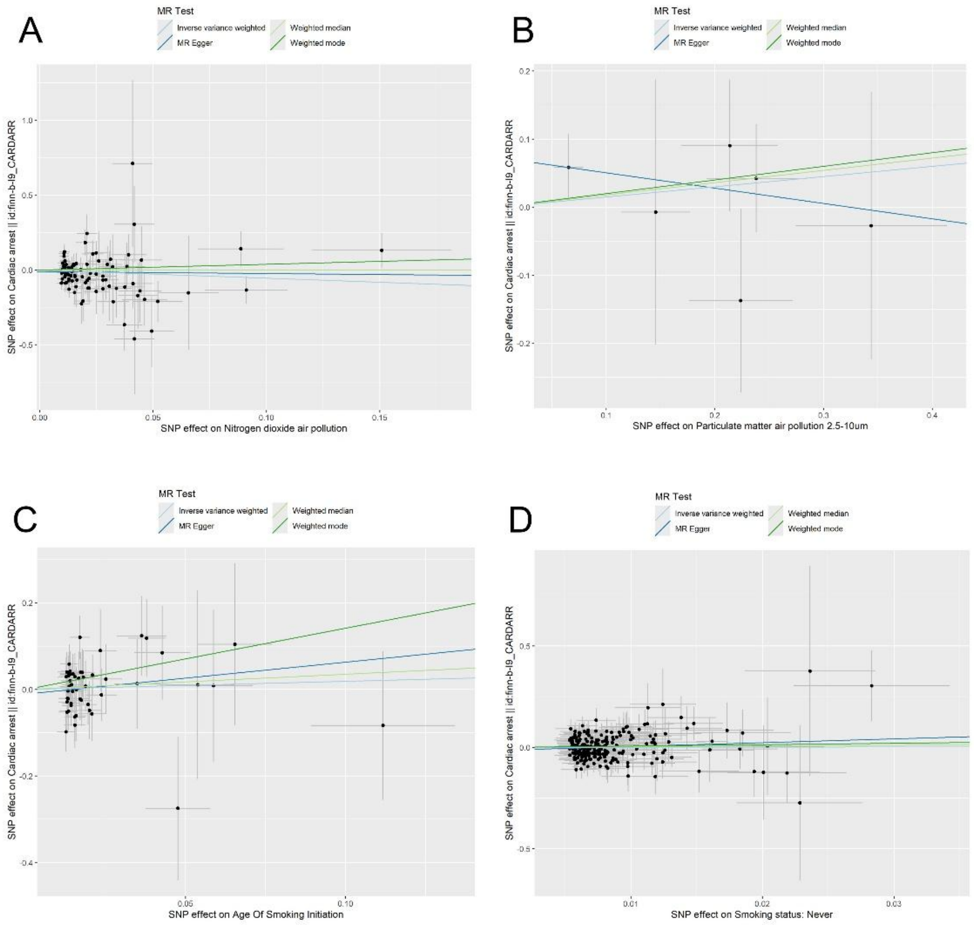

Figure S2. Scatter plots of the association between nitrogen dioxide air pollution(A), particulate matter air pollution 2.5-10um(B), age of smoking initiation(C), smoking status: never(D) and CA.

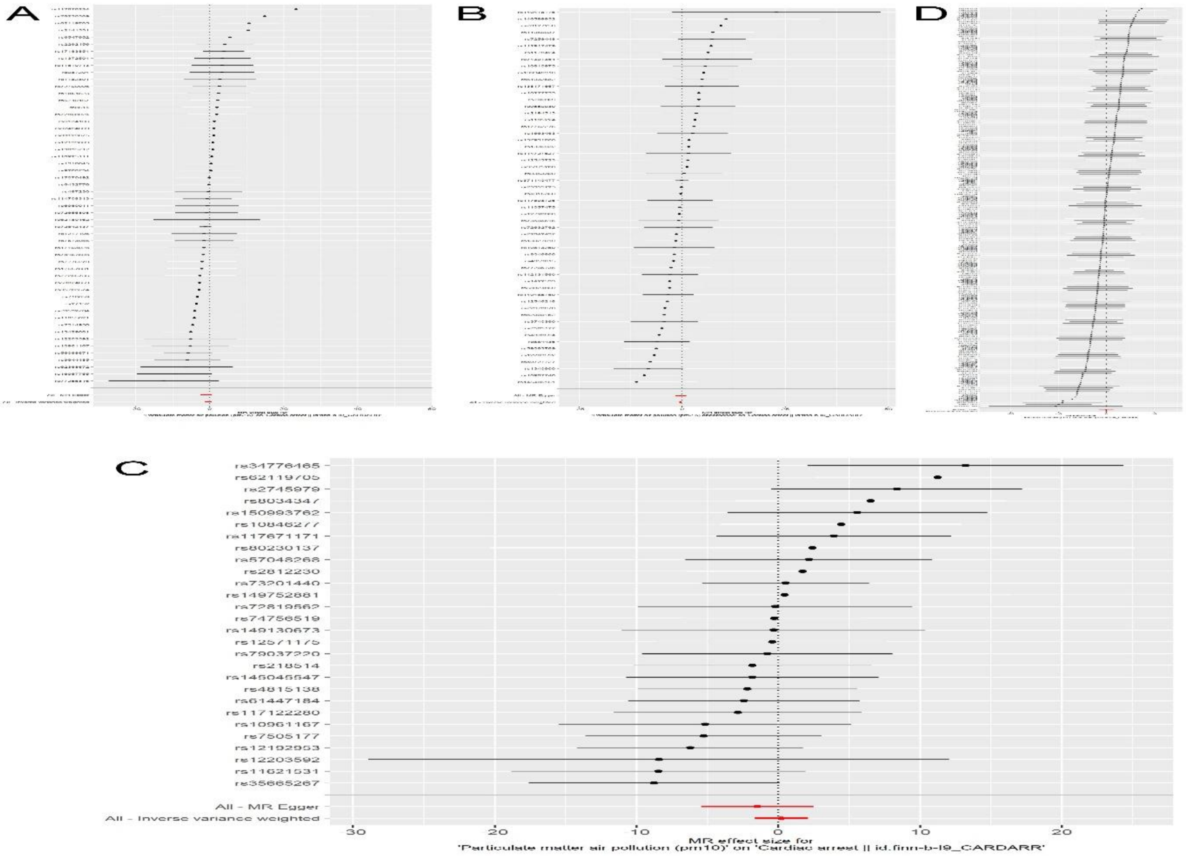

Figure S3. Forest plots of the association between particulate matter air pollution (PM2.5) (A), particulate matter air pollution (PM2.5) absorbance (B), particulate matter air pollution (PM10)(C), past tobacco smoking(D) and CA.

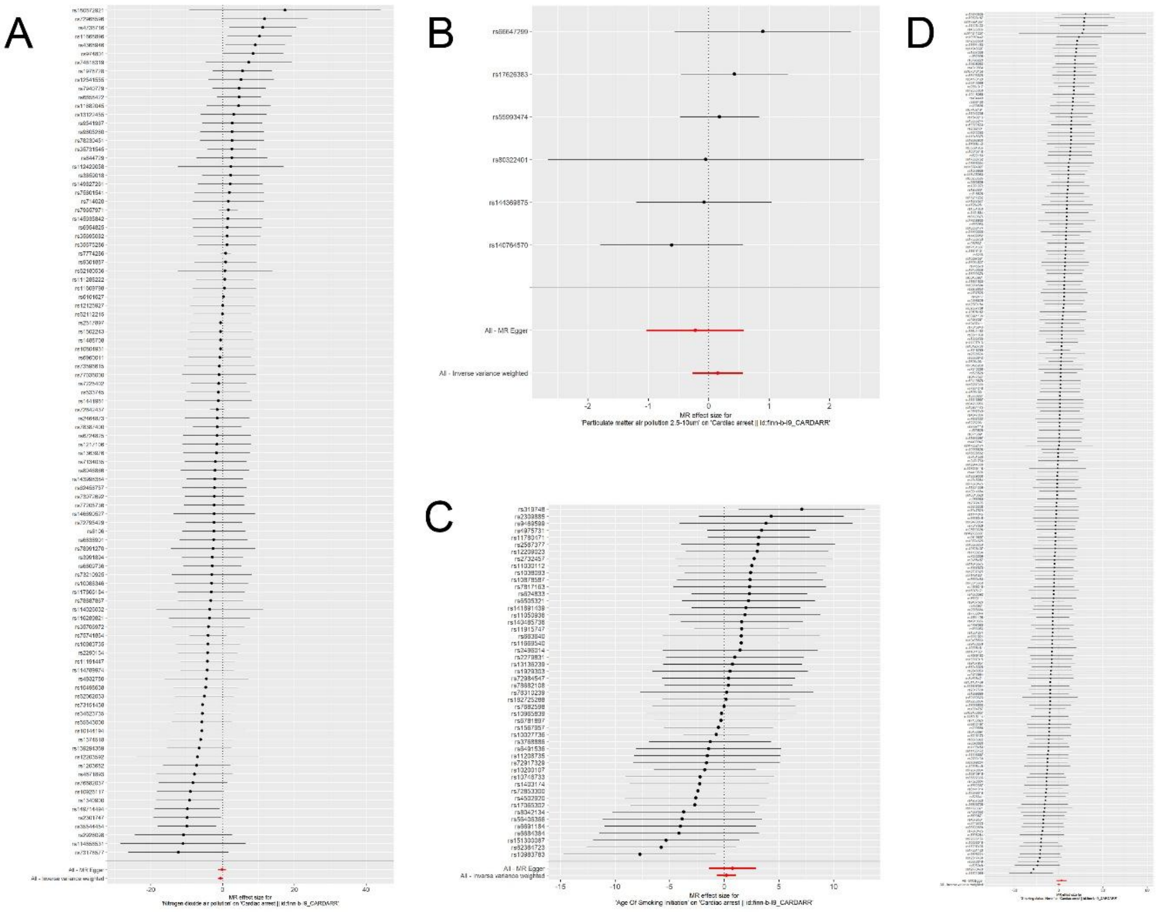

Figure S4. Forest plots of the association between nitrogen dioxide air pollution(A), particulate matter air pollution 2.5-10um(B), age of smoking initiation(C), and smoking status: never(D) and CA.

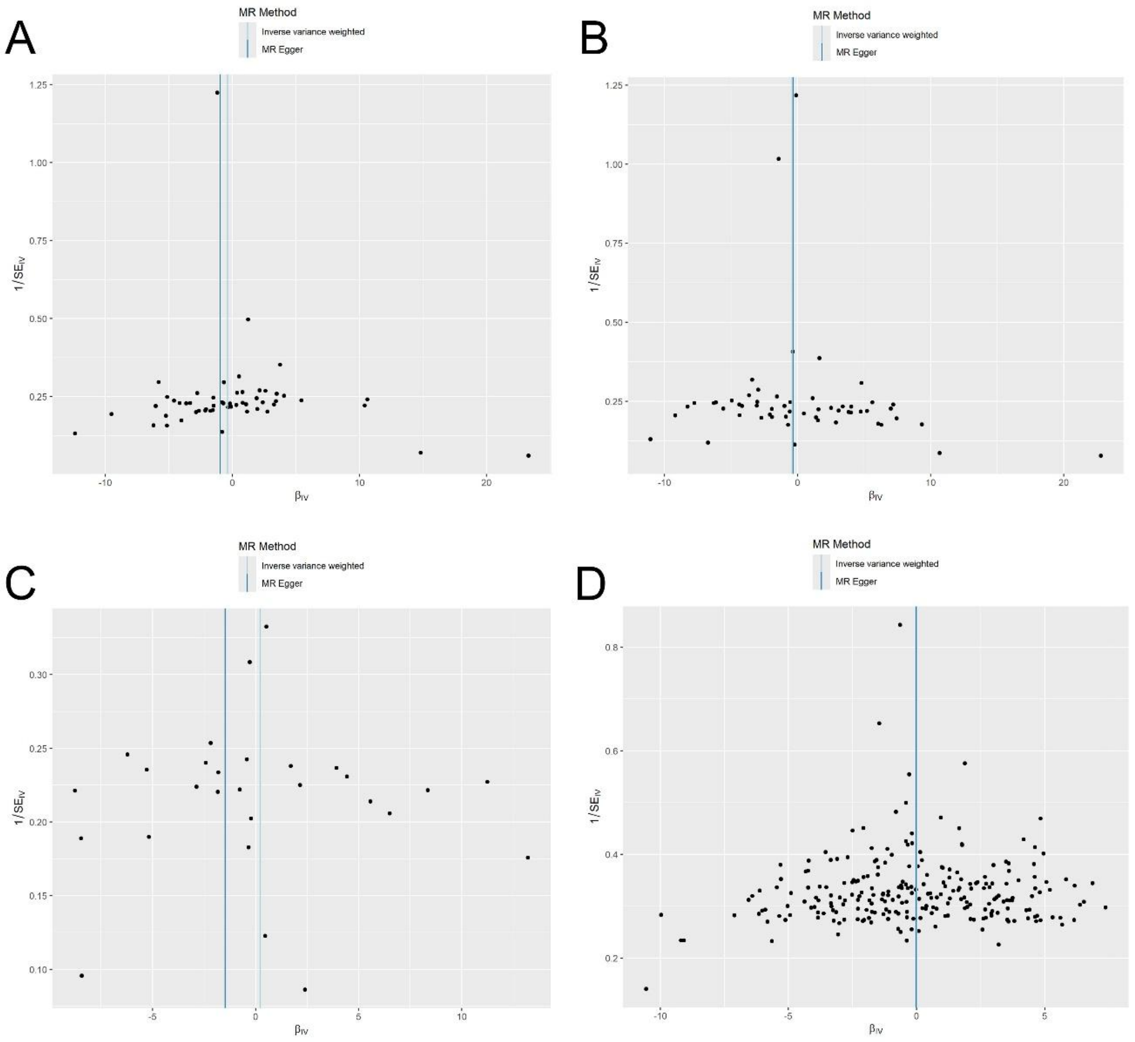

Figure S5. Funnel plots of the association between particulate matter air pollution (PM2.5) (A), particulate matter air pollution (PM2.5) absorbance (B), particulate matter air pollution (PM10)(C), past tobacco smoking(D) and CA.

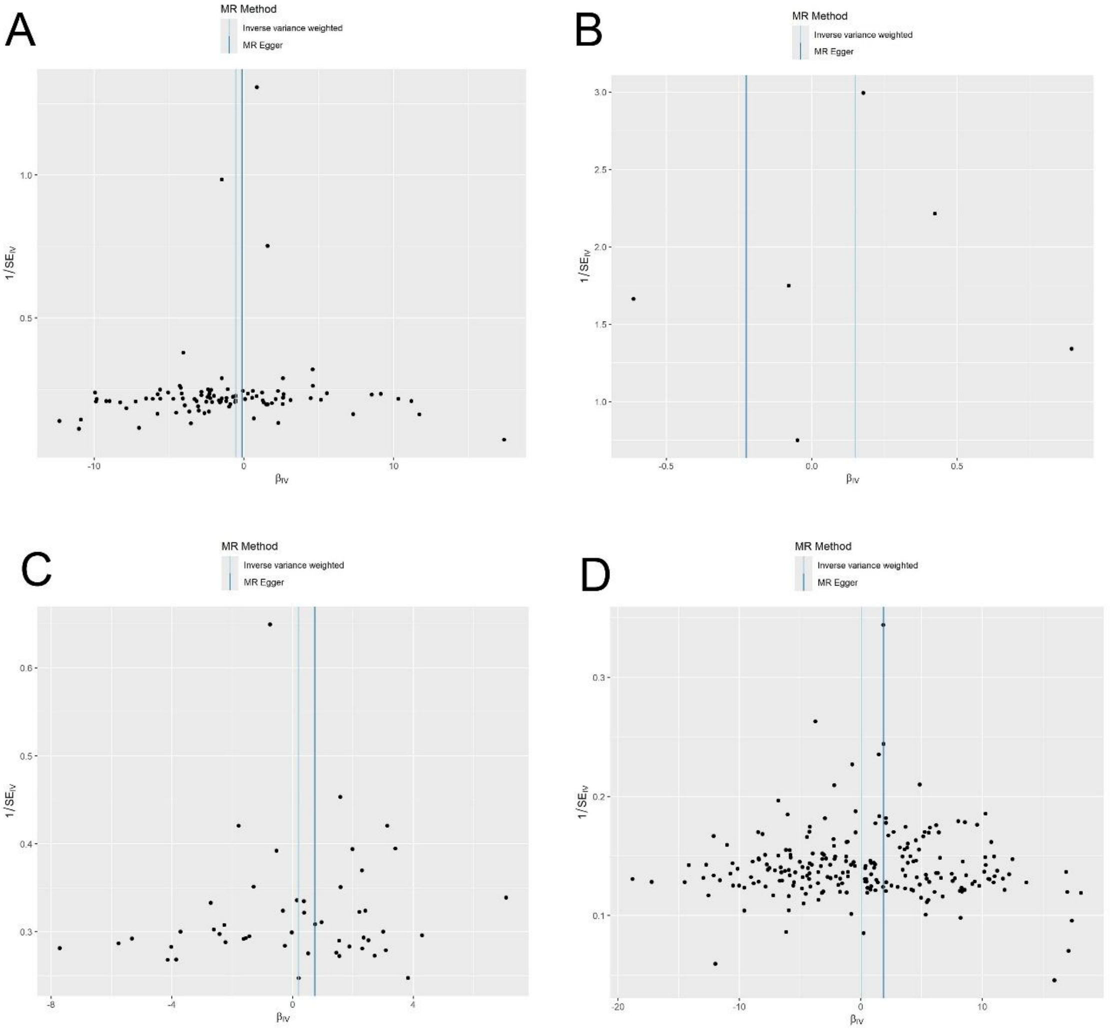

Figure S6. Funnel plots of the association between nitrogen dioxide air pollution(A), particulate matter air pollution 2.5-10um(B), age of smoking initiation(C), and smoking status: never(D) and CA.

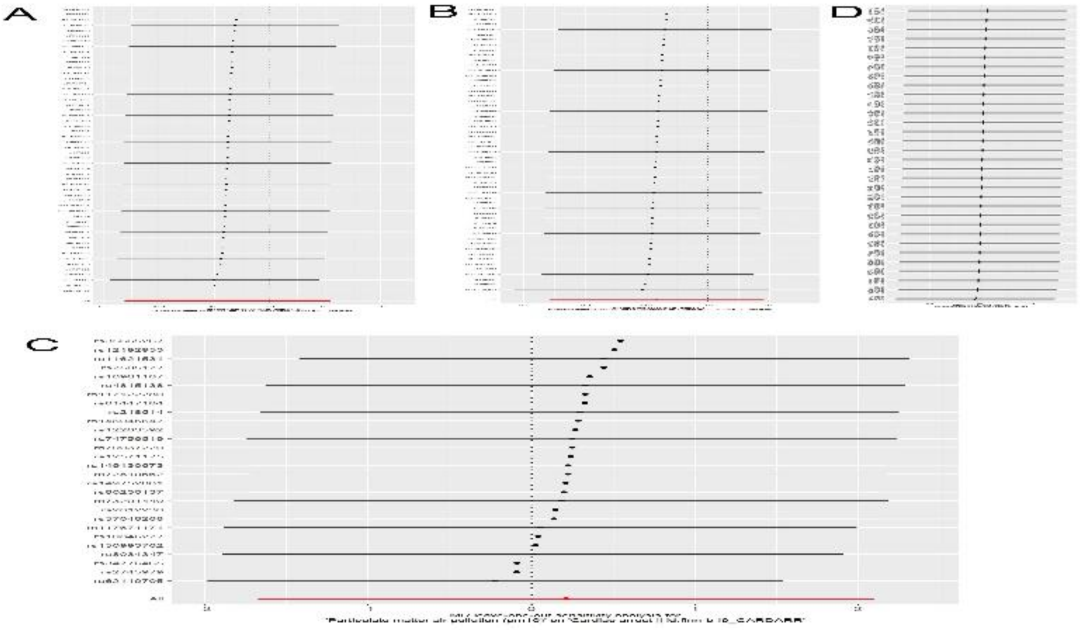

Figure S7. Leave-one-out plots of the association between particulate matter air pollution (PM2.5) (A), particulate matter air pollution (PM2.5) absorbance (B), particulate matter air pollution (PM10)(C), past tobacco smoking(D) and CA.

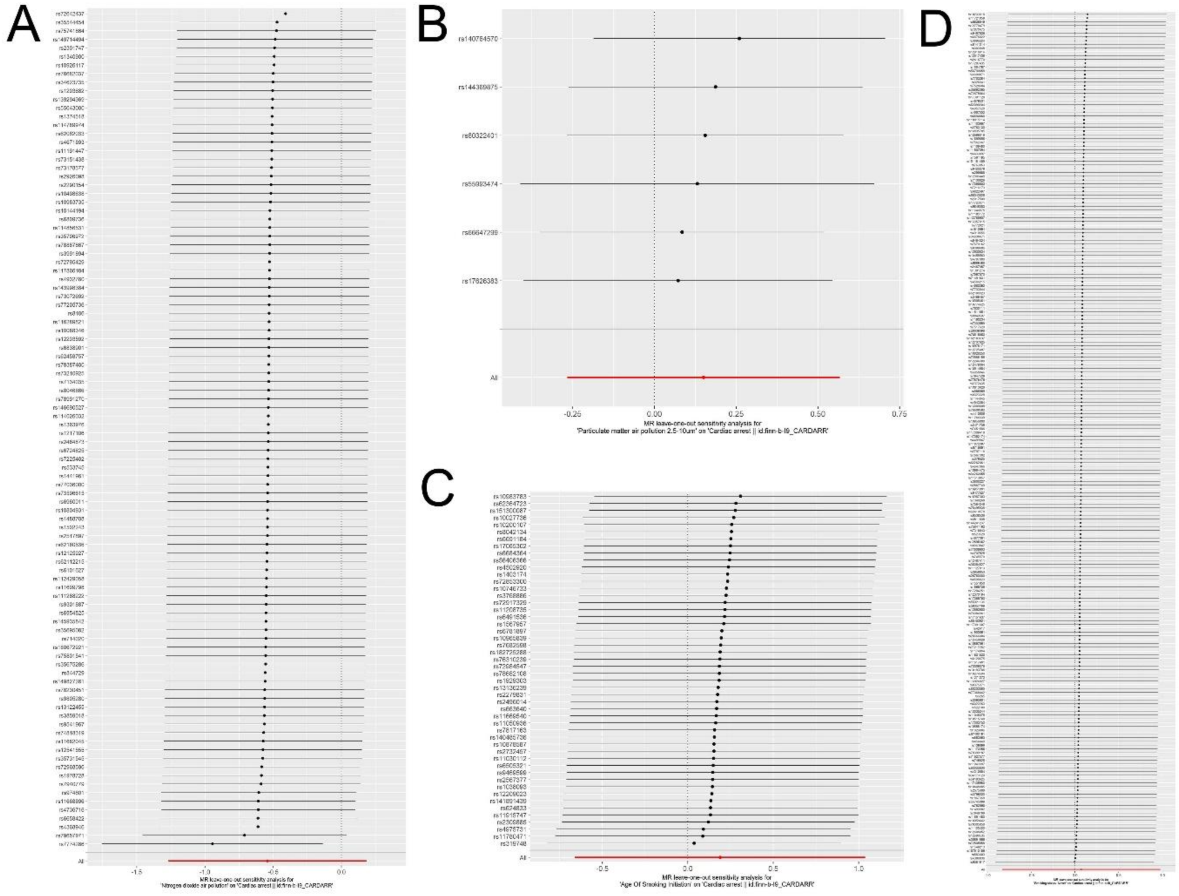

Figure S8. Leave-one-out plots of the association between nitrogen dioxide air pollution(A), particulate matter air pollution 2.5-10um(B), age of smoking initiation(C), and smoking status: never(D) and CA.
