# Supplementary material for: Um Estudo de Randomização Mendeliana de Duas Amostras sobre Poluição do Ar/Tabagismo e Parada Cardíaca
Source: Arq Bras Cardiol. 2026 Mar 26;123(3):20250127. [Article in Portuguese] doi: 10.36660/abc.20250127 (PMC13128207; doi:10.36660/abc.20250127)
Supplement: Tabela(s) suplementar(es) [file 0066-782x-abc-123-3-20250127-suppl2.pdf]

Table S1. Details on IV selection

Trait	GWAS ID	N（case/control）	No. of SNPs
Particulate matter air pollution (PM10)	ukb-b-18469	423,796	9,851,867
Particulate matter air pollution (PM2.5)	ukb-b-10817	423,796	9,851,867
Particulate matter air pollution (PM2.5) absorbance	ukb-b-11312	423,796	9,851,867
Nitrogen dioxide air pollution	ukb-b-2618	456,380	9,851,867
Particulate matter air pollution 2.5-10um	ukb-e-24008_CSA	8,567	9,811,988
Smoking status	ebi-a-GCST90029014	468,170	11,973,425
Past tobacco smoking	ukb-b-2134	424,960	9,851,867

Smoking status: Never	ukb-d-20116_0	195,068/164,638	13,586,591
Age of smoking Initiation	ieu-b-24	341,427	11,894,779
Cardiac arrest	finn-b-I9_CARDARR	118055	16,379,486

Table S2. Details on IV associated with air pollution and smoking

**Exposure**	**SNP**	**Mean F**	**Max F**	**Min F**
Age of smoking initiation	52	52.7	20.9	25.29231
Nitrogen dioxide air pollution	105	46.11	20.87	24.0799
Particulate matter air pollution (PM10)	29	28.06	21.12	23.13034
Particulate matter air pollution (PM2.5)	58	69.92	20.88	24.88241
Particulate matter air pollution (PM2.5) absorbance	58	41.54	20.87	23.99603
Particulate matter air pollution 2.5-10um	8	26.74	21.33	23.12375

Past tobacco smoking	258	203.02	20.86	31.0488
Smoking status	332	191.09	20.34	31.6291
Smoking status: Never	238	182.62	20.84	30.47202

Table S3. Details on IV associated with CA

**Target SNP**	**Proxy SNP**
rs72505558	rs896778
rs8085914	rs8095030
rs3958046	rs7777351
rs137889532	rs7742673
rs62262061	rs7635601
rs17774927	rs73097319
rs3768886	rs72974267
rs10105665	rs7000535
rs7004274	rs6988143
rs7340268	rs6748283
rs62484669	rs62484681
rs12370184	rs595348

rs12919081	rs461405
rs79478087	rs4333102
rs35923807	rs34435038
rs142785607	rs264921
rs6951574	rs2622226
rs68043026	rs2424244
rs6822497	rs2131464
rs6442644	rs2084533
rs12690535	rs2030199
rs1978728	rs1978727
rs79657971	rs185324621
rs62062033	rs17730540
rs76084961	rs17518584
rs73123076	rs13357015

rs56790858	rs13027140
rs11012750	rs1270799
rs9848053	rs11925445
rs11855821	rs11856579
rs138171657	rs117117437
rs11513135	rs11246496
rs10448085	rs111415834
rs12244388	rs10786724
rs1561195	rs10780649
rs2416770	rs1031111
rs10274594	rs10268837

Table S4. Pleiotropy and heterogeneity analyses

Exposure	Outcome	Heterogeneity			Pleiotropy	
		Q statistic (IVW)	*P* value		MR-Egger Intercept	*P* value
Smoking status	Cardiac arrest	326.155	0.335		0.0166	0.151
Age Of Smoking Initiation	Cardiac arrest	38.680	0.855		-0.0107	0.589
Particulate matter air pollution (PM2.5)	Cardiac arrest	79.988	0.012		0.0247	0.157
Particulate matter air pollution (PM2.5)	Cardiac arrest	58.533	0.347		-0.0024	0.863

absorbance						
Particulate matter air pollution (PM10)	Cardiac arrest	34.147	0.162		0.0339	0.348
Past tobacco smoking	Cardiac arrest	259.441	0.185		0.0007	0.956
Nitrogen dioxide air pollution	Cardiac arrest	102.589	0.329		-0.0103	0.348
Smoking status: Never	Cardiac arrest	202.568	0.856		-0.0142	0.280
Particulate matter air pollution 2.5-10um	Cardiac arrest	3.162	0.675		0.0731	0.347

Table S5. Pleiotropy analyses and outlier detection using the MR-PRESSO method

Exposure	Outcome	Raw			Outlier corrected		Global P	Number of outliers
		OR (CI%)	*P*		OR (CI%)	*P*		
Smoking status	Cardiac arrest	2.182 (1.137 , 4.188)	0.02		NA	NA	0.335	NA
Age Of Smoking Initiation	Cardiac arrest	1.208 (0.568 , 2.568)	0.626		NA	NA	0.849	NA
Past tobacco smoking	Cardiac arrest	1.007 (0.681 , 1.489)	0.972		NA	NA	0.192	NA
Smoking status: Never	Cardiac arrest	1.075 (0.456 , 2.534)	0.869		NA	NA	0.856	NA
Nitrogen dioxide air pollution	Cardiac arrest	0.582 (0.281 , 1.202)	0.147		NA	NA	0.291	NA

Particulate matter air pollution (PM2.5) absorbance	Cardiac arrest	0.658 (0.275 , 1.577)	0.353		NA	NA	0.357	NA
Particulate matter air pollution (PM10)	Cardiac arrest	1.234 (0.187 , 8.123)	0.829		NA	NA	0.197	NA
Particulate matter air pollution (PM2.5)	Cardiac arrest	0.973 (0.316 , 2.998)	0.962		0.683 (0.286 , 1.631)	0.395	0.016	1(rs1537371)
Particulate matter air pollution 2.5-10um	Cardiac arrest	1.162 (0.834 , 1.619)	0.416		NA	NA	0.734	NA

Table S6. Results of statistical power calculations

Exposure	Outcome	IVW_OR	Exposure_R2	N	K	Power
Age Of Smoking Initiation	Cardiac arrest	1.20757	0.005017	341427	0.009563	13.28%
Nitrogen dioxide air pollution	Cardiac arrest	0.58153	0.005175	456380	0.009563	51.16%
Particulate matter air pollution (pm10)	Cardiac arrest	1.233729	0.00153	423796	0.009563	8.91%
Particulate matter air pollution (pm2.5)	Cardiac arrest	0.972542	0.00324	423796	0.009563	5.11%
Particulate matter air pollution (pm2.5) absorbance	Cardiac arrest	0.658405	0.003148	423796	0.009563	23.01%
Particulate matter air pollution 2.5-10um	Cardiac arrest	1.162028	0.016476	8567	0.009563	5.40%
Past tobacco smoking	Cardiac arrest	1.007105	0.017524	424960	0.009563	5.04%
Smoking status	Cardiac arrest	2.182103	0.021279	468170	0.009563	100.00%
Smoking status: Never	Cardiac arrest	1.074862	0.019172	359706	0.009563	9.28%
