# Supplementary material for: Um Estudo de Randomização Mendeliana de Duas Amostras sobre Poluição do Ar/Tabagismo e Parada Cardíaca
Source: Arq Bras Cardiol. 2026 Mar 26;123(3):20250127. [Article in Portuguese] doi: 10.36660/abc.20250127 (PMC13128207; doi:10.36660/abc.20250127)
Supplement: Translation

# Translation: A Two-sample Mendelian Randomization Study of Air Pollution/Smoking and Cardiac Arrest

**DOI:** 10.36660/abc.20250127i

**Authors:** Xuelin Tan, Yu Wang, Qingzhao Tan, Liying Liu

**Affiliations:** 1 Zhongshan Hospital Xiamen University Xiamen China Zhongshan Hospital Xiamen University, Xiamen – China; 2 Xiamen University Department of Emergency Xiamen China Xiamen University - Department of Emergency, Xiamen – China

**Keywords:** Air Pollution, Tobacco Use Disorder, Heart Arrest, Mendelian Randomization Analysis

## Abstract

**Background:**

Observational studies have suggested a connection between air pollution/smoking and cardiac arrest (CA); nevertheless, the causal role of air pollution/smoking in CA remains uncertain.

**Objectives:**

This study aimed to elucidate the causal association between air pollution/smoking and CA using a two-sample Mendelian randomization (MR) approach.

**Methods:**

This study utilized summary data from genome-wide association studies (GWAS). The primary analysis employed the inverse-variance weighted (IVW) method, supplemented by MR-Egger, weighted median, and weighted mode methods. For sensitivity analysis, MR-Egger and MR-PRESSO were employed to address horizontal pleiotropy. Cochran’s Q test was used to assess heterogeneity, and leave-one-out analysis was applied to identify the influence of outliers. A two-sided p-value < 0.05 was considered statistically significant for the causal estimates.

**Results:**

IVW method showed a significant association between smoking status and CA (OR = 2.182, 95% CI: 1.137 – 4.188, p = 0.019). No significant causal associations were found for any of the air pollution metrics or other smoking-related traits. Results of MR Egger and weighted median methods revealed an insignificant causal association between air pollution/smoking and CA. Horizontal pleiotropy or outliers were not detected by sensitivity tests.

**Conclusions:**

Findings of this MR study indicate that smoking has a causal effect on the incidence of developing CA. However, the null findings for air pollution should be interpreted with caution, as the statistical power to detect modest effects was limited. More studies are warranted to verify the outcomes of this study.

## Introduction

Cardiac arrest (CA) signifies the failure of mechanical cardiac activity, coupled with the lack of observable clinical evidence of systemic circulation.^1^Globally, CA is responsible for an estimated 15% of all fatalities and comprises half, or 50%, of all cardiac-related deaths.^2^Differences in underlying pathophysiology and systems of care necessitate the examination of epidemiology through two distinct categories: in-hospital cardiac arrest and out-of-hospital cardiac arrest (OHCA).^1^OHCA, defined as the cessation of cardiac mechanical activity occurring outside a hospital setting, is the most common and critical condition marked by a low survival rate. Lifestyle factors, such as smoking, levels of physical activity, and diet, have also been found to be associated with CA.^3^In addition, growing concerns have led to the recognition of environmental factors as playing a crucial role in the initiation of OHCAs; consequently, the association between acute exposure to air pollutants and OHCA incidence has risen to prominence as a focal point of recent research.

In a prospective study examining the association between cigarette smoking and smoking cessation on the risk of SCD among 101,018 women, compared to never smokers, current smokers exhibited a 2.44-fold elevated risk of CA (95% confidence interval CI: 1.80 - 3.31), with adjustments made for coronary risk factors, In multifactorial analyses, both the daily quantity of cigarettes smoked (P for trend <0.0001) and the duration of smoking (P for trend <0.0001) demonstrated linear correlations with augmented CA risk among current smokers compared to an 8% rise in CA risk (hazard ratio (HR)=1.08; 95% CI=1.05–1.12; p < 0.0001).^4^A prior study has documented that smoking cessation can lead to a substantial decrease in the risk of CA.^5^In a multicenter case-control study at 17 hospitals in Korea, results showed that current smoking was associated with CA incidence (adjusted odds ratio (OR) = 1.39, 95% CI: 1.08–1.79).^6^In addition, epidemiological researches have consistently revealed an elevated risk of cardiovascular diseases and mortality linked to the presence of particulate matter (PM) in ambient air pollution.^7 , 8^Especially, previous studies have investigated the correlation between air pollution levels and the incidence of OHCA. The risk of OHCA has been correlated with short-term elevations in exposure to PM, specifically fine particles with an aerodynamic diameter less than 2.5 micrometers (PM2.5) or ultrafine PM, and at times, coarser particles with diameters less than 10 micrometers (PM10), along with gaseous pollutants such as ozone (O_3_), which can originate from various sources including industrial activities and vehicular emissions.^9^

Nonetheless, the findings from observational studies are susceptible to being influenced by confounding factors, notably from baseline characteristics and study design. Mendelian Randomization (MR) is a sophisticated instrumental variable (IV) strategy that employs single-nucleotide polymorphisms (SNPs) as inherent natural IVs, facilitating the dissection of causal relationships between phenotypic traits.^10^This methodology inherently mitigates biases arising from confounding variables and sidesteps the issue of reverse causality, thereby enhancing the credibility of inferred causal links. This study aimed to investigate the causal relationship between air pollution/smoking and the risk of CA. Findings of this study may assist in illuminating the genetic underpinnings and biological pathways implicated in CA.

## Methods

### Study design

The present MR study was conducted under three fundamental assumptions: (1) Relevance: The genetic variant is significantly associated with the exposure under investigation; (2) Independence: The genetic variant is independent of any confounding factors that might influence the relationship between the exposure and outcome; and (3) Exclusion Restriction: The genetic variant impacts the outcome solely through its connection with the exposure, exerting no direct independent effect.^11^This study employed a two-sample MR analysis to evaluate the causal association between air pollution/smoking and the risk of CA ( Central Illustration ).

### Data source

In this study, CA was regarded as the outcome, and air pollution and smoking were regarded as exposure. The summary-level data on CA were obtained from the FinnGen dataset. The sample size was 118055. Data on smoking were obtained from the IEU Open GWAS project (https://gwas.mrcieu.ac.uk/), and data on air pollution were obtained from the Medical Research Council- Integrative Epidemiology Unit (MRC-IEU).^12^Land use regression (LUR) model from the ESCAPE project was utilized to estimate concentrations of air pollutants in the vicinity of households, which were then integrated with genomic data from blood sequencing available in the UK Biobank (UKB) cohort dataset.^13 , 14^Detailed information is provided in Table S1 . All data are publicly available, and ethical approval and informed consent have been obtained in the original studies.

### Instrumental variables (IVs) selection

To adhere to the aforementioned assumptions, the GWAS database was queried for SNP selection. Subsequently, a significance threshold of P < 5 × 10^6^was used to select SNPs as potential instrumental variables (IVs), this slightly relaxed threshold, compared to the strict genome-wide significance level (P < 5 × 10^8^), is a commonly employed strategy in MR studies to increase the number of available instruments, thereby enhancing the statistical power of the analysis, especially for exposures with a limited number of genome-wide significant hits.^15^The inclusion criterion for the minor allele frequency (MAF) of the variants under investigation was set at 0.01.^10^All identified SNPs were subjected to clumping to preclude linkage disequilibrium issues, employing a stringent clump window parameter set at an R^2^threshold of 0.001 and a kb of 10,000.^9^When instrumental variables were absent in the outcome dataset, high-linkage disequilibrium (LD) proxies, with an R^2^exceeding 0.8, were sought out and utilized as replacements.^16^For assessing instrument strength and to address potential weak instrument bias, the F-statistic was calculated for every SNP within the IV set. This computation adhered to the formula: $F=\left[R^{2}*(N-2)\right]/\left(1-R^{2}\right)$ , wherein R^2^signifies the proportion of variance in exposure explained by the SNP, and N represents the sample size.^17^To ensure robust instrument potency and avoid weak instrument bias, only SNPs yielding an F-statistic > 10 were retained.^17^

### Mendelian Randomization

The two-sample MR analysis was conducted utilizing the R software, version 4.3.3, employing the TwoSampleMR package and the MR-PRESSO package. The primary MR analysis was executed employing the Inverse-Variance Weighted (IVW) method, premised on the assumption that no average pleiotropic effects are present. The IVW method is considered the most effective approach for estimating the causal link between air pollution/smoking and CA, utilizing ORs alongside their respective 95% CIs to quantify the association. To assess the robustness of our findings, additional analytical methods, including MR-Egger regression, weighted median, and weighted mode approaches, were employed as complementary analyses. The MR-Egger approach incorporates an intercept term, allowing for the provision of unbiased estimates of causal effects even when horizontal pleiotropy is present.^18^The weighted median approach is predicated on the assumption that at least half of the instrumental variables accurately estimate the causal relationship between the exposure and the outcome.^19^The weighted mode strategy identifies the causal effect by focusing on a subset of SNPs that exhibit the highest density of effects, accomplished by clustering SNPs into groups according to their similarity in inferred causal impacts.^20^Multiple testing adjustments were carried out employing the Benjamini-Hochberg false discovery rate (FDR) procedure, taking into account the effective number of independent traits.^21^A scatter plot was used to depict the association between air pollution/smoking and CA. A forest plot was employed to show the MR analysis and 95% CI for each SNP.

### Sensitivity analysis

Sensitivity analyses were conducted to verify the reliability of MR estimates. In the IVW analysis, the I^2^statistic and Cochran’s Q test were employed to assess heterogeneity among the effects of SNPs associated with CA outcomes, with a p-value greater than 0.05 indicating no statistically significant heterogeneity.^22^The MR-Egger regression is employed to detect potential pleiotropy and assess its impact on risk estimation, with the intercept test being pivotal. A p-value > 0.05 suggests an absence of significant pleiotropy.^23^A funnel plot was utilized to visually inspect and illustrate the presence of horizontal pleiotropy. Moreover, the MR Pleiotropy Residual Sum and Outlier (MR-PRESSO) method was employed to systematically assess horizontal pleiotropy and to identify and subsequently remove any outlier SNPs that could distort the causal inference.^24^Furthermore, after excluding outlier instrumental variables (IVs), we examined whether there was a statistically significant difference in the results compared to before their removal. A leave-one-out (LOO) analysis was conducted to ascertain the influence of each SNP on the overall causal relationship, thus ensuring the robustness of our findings.

### Statistical power

The statistical power of our MR analysis was assessed using the online tool “mRnd” (https://shiny.cnsgenomics.com/mRnd/), which is specifically designed for power calculations in IV analyses. These calculators estimate the power to detect an effect of a given magnitude for a continuous outcome, based on several key parameters: (1) the proportion of variance in the exposure explained by the IV (R^2^), (2) the true causal effect size, (3) the sample size of the outcome GWAS, (4) the number of genetic instruments used, and (5) the pre-specified significance level (α).^25^

## Results

### IVs selection

This study identified 1138 IVs associated with air pollution and smoking. Upon calculating the F-statistics for these IVs, the mean F-statistic was 29.26. Importantly, the minimum F-statistic for any included SNP was 20.34, which is substantially higher than the conventional threshold of 10, indicating that our analyses are unlikely to be affected by weak instrument bias ( Table S2 ). In the MR analysis with CA as the endpoint, 56 SNPs were unmatched in the summary data, and 19 SNPs lacked appropriate proxy SNPs ( Table S3 ).

### Mendelian Randomization

Results of IVW showed a significant association between smoking status and CA (OR = 2.182, 95% CI: 1.137 – 4.188, p = 0.019). Figures 1 and 2 depict the scatter plots and forest plots for the effect of smoking status on CA. In addition, the IVW results showed no evidence of a causal relationship between various air pollution metrics (including PM2.5, PM2.5 absorbance, PM10, and nitrogen dioxide) or other smoking-related traits (past tobacco smoking, age of initiation, and never smoking status) and the risk of CA. The detailed results for these analyses are presented in Table 1 . The scatter plot for the negative results is shown in Figures S1 -S2 , and the forest plot for the negative results is shown in Figures S3 –S4 .

**Figure 1:** Scatter plots of the association between Smoking status and CA. The slopes depicted the causal associations, with each line representing a different methodology.

**Figure 2:** Forest plots of the association between Smoking status and CA. The MR estimate derived from the MR-Egger and IVW methods was shown for comparison.

**Table 1:** Results of the causal relationship between air pollution/smoking and CA

Exposure	Outcome	Significance of SNP	N. SNPs	Methods	OR (95% CI)	P
Smoking status	Cardiac arrest	5*10^-6^	317	MR Egger	0.374 ( 0.031, 4.501 )	0.439
Smoking status	Cardiac arrest		317	Weighted median	1.793 ( 0.669, 4.804 )	0.245
Smoking status	Cardiac arrest		317	Inverse variance weighted	2.182 ( 1.137, 4.188 )	0.019
Smoking status	Cardiac arrest		317	Weighted mode	0.673 ( 0.039, 11.63 )	0.786
Age Of Smoking Initiation	Cardiac arrest		50	MR Egger	2.091 (0.243, 17.968 )	0.505
Age Of Smoking Initiation	Cardiac arrest		50	Weighted median	1.424 ( 0.439, 4.622 )	0.556
Age Of Smoking Initiation	Cardiac arrest		50	Inverse variance weighted	1.208 ( 0.517, 2.823 )	0.663
Age Of Smoking Initiation	Cardiac arrest		50	Weighted mode	4.125 ( 0.355, 47.936 )	0.263
Particulate matter air pollution (PM2.5)	Cardiac arrest		55	MR Egger	0.383 ( 0.081, 1.81 )	0.231
Particulate matter air pollution (PM2.5)	Cardiac arrest		55	Weighted median	0.386 ( 0.078, 1.909 )	0.243
Particulate matter air pollution (PM2.5)	Cardiac arrest		55	Inverse variance weighted	0.683 ( 0.269, 1.736 )	0.424
Particulate matter air pollution (PM2.5)	Cardiac arrest		55	Weighted mode	0.435 ( 0.092, 2.051 )	0.298
Particulate matter air pollution (PM2.5) absorbance	Cardiac arrest		56	MR Egger	0.717 ( 0.193, 2.658 )	0.621
Particulate matter air pollution (PM2.5) absorbance	Cardiac arrest		56	Weighted median	0.819 ( 0.2, 3.358 )	0.781
Particulate matter air pollution (PM2.5) absorbance	Cardiac arrest		56	Inverse variance weighted	0.658 ( 0.275, 1.577 )	0.348
Particulate matter air pollution (PM2.5) absorbance	Cardiac arrest		56	Weighted mode	0.547 ( 0.157, 1.906 )	0.348
Particulate matter air pollution (PM10)	Cardiac arrest		28	MR Egger	0.228 ( 0.004, 11.78 )	0.469
Particulate matter air pollution (PM10)	Cardiac arrest		28	Weighted median	0.73 ( 0.071, 7.477 )	0.791
Particulate matter air pollution (PM10)	Cardiac arrest		28	Inverse variance weighted	1.234 ( 0.187, 8.123 )	0.827
Particulate matter air pollution (PM10)	Cardiac arrest		28	Weighted mode	0.53 ( 0.012, 24.257 )	0.747
Past tobacco smoking	Cardiac arrest		241	MR Egger	0.967 ( 0.214, 4.373 )	0.965
Past tobacco smoking	Cardiac arrest		241	Weighted median	0.747 ( 0.414, 1.348 )	0.333
Past tobacco smoking	Cardiac arrest		241	Inverse variance weighted	1.007 ( 0.681, 1.489 )	0.972
Past tobacco smoking	Cardiac arrest		241	Weighted mode	0.508 ( 0.116, 2.221 )	0.369
Nitrogen dioxide air pollution	Cardiac arrest		98	MR Egger	0.877 ( 0.286, 2.694 )	0.819
Nitrogen dioxide air pollution	Cardiac arrest		98	Weighted median	0.996 ( 0.302, 3.289 )	0.995
Nitrogen dioxide air pollution	Cardiac arrest		98	Inverse variance weighted	0.582 ( 0.281, 1.202 )	0.143
Nitrogen dioxide air pollution	Cardiac arrest		98	Weighted mode	1.473 ( 0.458, 4.741 )	0.518
Smoking status: Never	Cardiac arrest		226	MR Egger	6.496 (0.221, 191.07 )	0.279
Smoking status: Never	Cardiac arrest		226	Weighted median	1.433 ( 0.39, 5.267 )	0.588
Smoking status: Never	Cardiac arrest		226	Inverse variance weighted	1.075 ( 0.435, 2.654 )	0.876
Smoking status: Never	Cardiac arrest		226	Weighted mode	1.996 (0.054, 73.131 )	0.707
Particulate matter air pollution 2.5.-10um	Cardiac arrest		6	MR Egger	0.798 ( 0.356, 1.79 )	0.614
Particulate matter air pollution 2.5.-10um	Cardiac arrest		6	Weighted median	1.198 ( 0.698, 2.057 )	0.513
Particulate matter air pollution 2.5.-10um	Cardiac arrest		6	Inverse variance weighted	1.162 ( 0.766, 1.764 )	0.481
Particulate matter air pollution 2.5.-10um	Cardiac arrest		6	Weighted mode	1.222 ( 0.647, 2.306 )	0.564

### Sensitivity analyses

The MR-Egger regression analysis indicated an absence of bias due to horizontal pleiotropy ( Table S4 ). Heterogeneity was detected in the analysis linking particulate matter air pollution (PM2.5) to CA, whereas no heterogeneity was observed in the remaining analyses ( Table S4 ). Figures 3 and 4 depict the Funnel plots and Leave-one-out plots of the effect of smoking status on CA. Figures S5-S6 depict the Funnel plots of the negative results, and Figures S7-S8 depict the Leave-one-out plots of the negative results. According to the MR-PRESSO analysis, one outlier SNP (rs1537371) was identified in the PM2.5-CA association; however, even upon exclusion of this SNP, no statistically significant association persisted. No other outliers were detected in the rest of the analyses ( Table S5 ).

**Figure 3:** Funnel plots of the association between Smoking status and CA. Each point represents a genetic variant, plotted according to the estimated effect size and standard error. The plot helps identify asymmetry, which could indicate potential biases such as pleiotropy or publication bias.

**Figure 4:** Leave-one-out plots of the association between Smoking status and CA. Each point indicates the estimated effect size when one genetic variant is removed from the analysis. The horizontal lines represent the 95% confidence intervals, while the vertical line shows the overall pooled effect estimate.

### Statistical power

The statistical power calculations for our MR analyses are presented in Table S6 . The results indicate a generally low statistical power (< 80%) to detect causal effects for the majority of the exposure-outcome associations investigated.

## Discussion

To the best of our knowledge, this is the first MR analysis examining whether air pollution/smoking has a causal role in the development of CA. Findings of this study uncovered a causal connection between a genetic predisposition to smoking and CA. Additionally, our study has identified that air pollution was not causally related to CA.

Numerous investigations have assessed the association between SCA risk and adherence to healthy lifestyle practices. Chief among these factors, smoking stands out as the most recurrently examined lifestyle attribute in relation to CA.^5 , 26^In a prospective cohort study comprising women who were free of coronary heart disease at baseline, a robust dose-response relationship was uncovered between cigarette smoking and the risk of CA. Moreover, it was demonstrated that smoking cessation led to a substantial reduction and ultimately eradicated the elevated risk of CA.^4^Although the results were in line with the current study, the enrolled population was limited to women. In a case-control study by Park et al., non-smoking was proven to be a protective factor for CA risk (OR = 0.36, 95% CI: 0.27–0.47) when age, sex, residential area, cardiovascular comorbidities, and healthy lifestyle habits were adjusted.^27^Moreover, in a recent meta-analysis including 12 prospective studies, the pooled relative risk (RR) was estimated at 3.06 (95% CI: 2.46 – 3.82) for individuals who currently smoke, in comparison to those who have never smoked.^28^Approximately 80% of CA can be attributed to ventricular tachyarrhythmias, and a heightened vulnerability to both initial and recurrent incidents of these arrhythmias, as well as ventricular fibrillation among smokers, may be attributed to disturbances in ventricular repolarization time dispersion indices.^29 - 31^Nicotine, a key component of cigarettes, has been demonstrated in animal studies to provoke a spectrum of cardiac rhythm disorders, encompassing transient sinus pauses, bradycardia, sinus tachycardia, atrial fibrillation, sinoatrial block, atrioventricular block, and ventricular tachyarrhythmias, further implicating its role in the pathophysiology of smoking-related arrhythmogenic risk.^32^Findings of this study reinforced the importance of population-wide policies and strategies for smoking cessation in the reduction of the incidence of sudden cardiac deaths.

In previous observational studies, concentrations of air pollutants including PM2.5, PM10, NO_2_, and O_3_have been manifested as risk factors for CA.^26^Kang et al. conducted an analysis to assess the effects of elevated exposure to PM10 and PM2.5 on the occurrence of OHCA in a population setting. Their findings revealed a significant correlation between higher levels of PM2.5 exposure and an augmented risk of OHCA.^33^A study conducted in Stockholm, Sweden, revealed a notable exposure-response relationship, associating higher OHCA risk with increased O_3_levels, whereas no such association was observed for PM2.5 or NO_2_.^34^Conversely, a separate OHCA study undertaken in Lombardy, Italy, employing distinct methodologies, demonstrated that concentrations of all studied pollutants were significantly elevated on days with a high incidence of OHCA, except for O_3_.^35^A recent systematic review and meta-analysis encompassing 15 studies that investigated the relationship between OHCA and exposure to various atmospheric pollutants, with a primary focus on the pollutant concentrations of PM10, PM2.5, NO_2_, and O_3_, their analysis revealed a significant linkage between heightened OHCA risk and elevated levels of PM2.5, PM10, and O_3_in the ambient air.^36^Similarly, another study by Sielski et al. also reported on the influence of PM2.5 and PM10 on the parameters of out-of-hospital CA.^37^Disparity in some of the results from the aforementioned studies may be attributed to the different study designs, characteristics of participants, methodology of pollutant measurement, mixtures of exposures, and degrees of individual susceptibility. Of note, results of our MR analyses revealed an insignificant causal relation between air pollutants including PM10, PM2.5, NO_2_, and Particulate matter air pollution 2.5-10 um.

A primary consideration when interpreting these null associations is the statistical power of our analysis. As our power calculations indicate ( Table S6 ), the study was generally underpowered to detect small to moderate causal effects for air pollution metrics. Therefore, the absence of a statistically significant association should not be interpreted as definitive evidence of no causal effect, but rather may reflect the limitations of the current study to detect such a relationship. The underlying mechanisms for the insignificant findings with regard to the causal relationship between air pollution and CA could be that variations in the magnitude or character of the inflammatory response may emerge due to differences in particulate chemistry and the duration or intensity of exposures. Some individuals are likely more vulnerable, with evidence suggesting that those with underlying cardiovascular risks and metabolic syndrome may demonstrate stronger associations.^37^Conversely, anti-inflammatory medications, like statins, might counteract the effects of ambient particles.^30^The magnitude and consistency of these changes vary across a range of biomarkers and patient populations studied.^8^It is important to note that MR estimates the causal effect of lifelong, genetically predicted exposure levels. In contrast, many observational studies have linked CA to acute, short-term spikes in air pollution. It is plausible that chronic, lower-level exposure (as proxied by genetics) does not have a detectable causal effect, whereas acute high-level exposure acts as a trigger for cardiac events in susceptible individuals. Our study was not designed to capture such short-term effects. Furthermore, the relationship between air pollution and CA might be non-linear, with risk increasing significantly only above a certain exposure threshold not captured by the genetic instruments. While MR is robust against many confounding factors, we cannot completely rule out unmeasured mediators or pathways of horizontal pleiotropy. For example, socioeconomic status is a complex confounder linked to both residential air pollution levels and cardiovascular health, and its effects may not be fully captured.

Our study boasts several strengths. It contributes valuable insights to the understanding of the causal relationship between air pollution/smoking and CA, offering empirical grounding for further research in this domain. Additionally, by challenging the notion that cigarette smoking could be a dependable risk factor for CA, our findings carry important implications. However, we acknowledge several limitations of this study. Primarily, our analysis was confined to cohorts of European descent, which limits the generalizability of our findings. Genetic architecture, such as allele frequencies and linkage disequilibrium patterns, can differ across populations. Moreover, gene-environment interactions may vary; for instance, the impact of genetic predisposition to smoking could be modified by different social norms or exposure levels to other risk factors in non-European populations. Therefore, future MR studies in diverse ancestral groups, such as those of African or Asian descent, are crucial to validate our findings and assess their global public health implications. Secondly, though we cannot definitively exclude participant overlap between datasets for exposure and outcome, quantifying this overlap accurately poses a methodological challenge. Despite this, the high robustness of our IVs, as evidenced by F statistics > 10, significantly mitigates potential bias arising from any such overlap, aligning with established criteria.^17^Thirdly, a significant limitation is the insufficient statistical power for several of our analyses, particularly for air pollution exposures. This limits our ability to draw firm conclusions from the null findings and highlights that the absence of evidence is not evidence of absence. This issue underscores the need for future studies using larger GWAS summary datasets, which would provide greater power to detect potential small to moderate causal effects. Given these limitations, cautious interpretation of the outcomes of this study is warranted, underscoring the need for future studies that address these constraints and expand the scope of investigation to broader populations and with a more comprehensive set of SNPs to enhance the precision and generalizability of findings.

## Conclusions

The results from this two-sample MR analysis provide evidence for a genetically inferred causal link between cigarette smoking and CA. Specific strategies for smoking cessation are needed to reduce the occurrence of CA. To solidify outcomes of the current study, future analyses should employ larger GWAS summary datasets based on multiple populations and an expanded repertoire of genetic instruments.

## Supplemental Materials

Supplementary Figure(s)

Supplementary Table(s)

## References

[1] Gill R, Teitcher M, Ruland S (2021). Handb Clin Neurol.

[2] Albert CM, Stevenson WG, Jameson JL, Fauci AS, Kasper DL, Hauser SL, Longo DL, Loscalzo J (2018). Harrison's Principles of Internal Medicine.

[3] Chiuve SE, Fung TT, Rexrode KM, Spiegelman D, Manson JE, Stampfer MJ (2011). Adherence to a Low-Risk, Healthy Lifestyle and Risk of Sudden Cardiac Death among Women. JAMA.

[4] Sandhu RK, Jimenez MC, Chiuve SE, Fitzgerald KC, Kenfield SA, Tedrow UB (2012). Smoking, Smoking Cessation, and Risk of Sudden Cardiac Death in Women. Circ Arrhythm Electrophysiol.

[5] Goldenberg I, Jonas M, Tenenbaum A, Boyko V, Matetzky S, Shotan A (2003). Current Smoking, Smoking Cessation, and the Risk of Sudden Cardiac Death in Patients with Coronary Artery Disease. Arch Intern Med.

[6] Jung E, Park JH, Ro YS, Ryu HH, Cha KC, Do Shin S (2023). Family History, Socioeconomic Factors, Comorbidities, Health Behaviors, and the Risk of Sudden Cardiac Arrest. Sci Rep.

[7] Brook RD, Franklin B, Cascio W, Hong Y, Howard G, Lipsett M (2004). Air Pollution and Cardiovascular Disease: A Statement for Healthcare Professionals from the Expert Panel on Population and Prevention Science of the American Heart Association. Circulation.

[8] Brook RD, Rajagopalan S, Pope CA, Brook JR, Bhatnagar A, Diez-Roux AV (2010). Particulate Matter air Pollution and Cardiovascular Disease: An Update to the Scientific Statement from the American Heart Association. Circulation.

[9] Abecasis GR, Altshuler D, Auton A, Brooks LD, Durbin RM, Gibbs RA (2010). A Map of Human Genome Variation from Population-Scale Sequencing. Nature.

[10] Long Y, Tang L, Zhou Y, Zhao S, Zhu H (2023). Causal Relationship between Gut Microbiota and Cancers: A Two-Sample Mendelian Randomisation Study. BMC Med.

[11] Burgess S, Smith GD, Davies NM, Dudbridge F, Gill D, Glymour MM (2023). Guidelines for Performing Mendelian Randomization Investigations: Update for Summer 2023. Wellcome Open Res.

[12] Battram T, Yousefi P, Crawford G, Prince C, Sheikhalil Babaei M, Sharp G (2022). The EWAS Catalog: A Database of Epigenome-Wide Association Studies. Wellcome Open Res.

[13] Montagne D, Hoek G, Nieuwenhuijsen M, Lanki T, Pennanen A, Portella M (2014). The Association of LUR Modeled PM2.5 Elemental Composition with Personal Exposure. Sci Total Environ.

[14] Sudlow C, Gallacher J, Allen N, Beral V, Burton P, Danesh J (2015). UK Biobank: An Open Access Resource for Identifying the Causes of a Wide Range of Complex Diseases of Middle and Old Age. PLoS Med.

[15] Ference BA, Majeed F, Penumetcha R, Flack JM, Brook RD (2015). Effect of Naturally Random Allocation to Lower Low-Density Lipoprotein Cholesterol on the Risk of Coronary Heart Disease Mediated by Polymorphisms in NPC1L1, HMGCR, or Both: A 2 × 2 Factorial Mendelian Randomization Study. J Am Coll Cardiol.

[16] Deng MC, Liu F, Liang Y, Wang K, Nie JQ, Liu J (2023). Association between Frailty and Depression: A Bidirectional Mendelian Randomization Study. Sci Adv.

[17] Burgess S, Thompson SG, CRP CHD Genetics Collaboration (2011). Avoiding Bias from Weak Instruments in Mendelian Randomization Studies. Int J Epidemiol.

[18] Bowden J, Smith GD, Burgess S (2015). Mendelian Randomization with Invalid Instruments: Effect Estimation and Bias Detection Through Egger Regression. Int J Epidemiol.

[19] Bowden J, Smith GD, Haycock PC, Burgess S (2016). Consistent Estimation in Mendelian Randomization with Some Invalid Instruments Using a Weighted Median Estimator. Genet Epidemiol.

[20] Hartwig FP, Smith GD, Bowden J (2017). Robust Inference in Summary data Mendelian Randomization Via the Zero Modal Pleiotropy Assumption. Int J Epidemiol.

[21] Benjamini Y, Hochberg Y (2018). Controlling the False Discovery Rate: A Practical and Powerful Approach to Multiple Testing. J R Statist Soc B.

[22] Greco M FD, Minelli C, Sheehan NA, Thompson JR (2015). Detecting Pleiotropy in Mendelian Randomisation Studies with Summary Data and a Continuous Outcome. Stat Med.

[23] Burgess S, Thompson SG (2017). Interpreting Findings from Mendelian Randomization Using the MR-Egger Method. Eur J Epidemiol.

[24] Verbanck M, Chen CY, Neale B, Do R (2018). Detection of Widespread Horizontal Pleiotropy in Causal Relationships Inferred from Mendelian Randomization between Complex Traits and Diseases. Nat Genet.

[25] Brion MJ, Shakhbazov K, Visscher PM (2013). Calculating Statistical Power in Mendelian Randomization Studies. Int J Epidemiol.

[26] Adabag AS, Luepker RV, Roger VL, Gersh BJ (2010). Sudden Cardiac Death: Epidemiology and Risk Factors. Nat Rev Cardiol.

[27] Park JH, Cha KC, Ro YS, Song KJ, Shin SD, Jung WJ (2022). Healthy Lifestyle Factors, Cardiovascular Comorbidities, and the Risk of Sudden Cardiac Arrest: A Case-Control Study in Korea. Resuscitation.

[28] Aune D, Schlesinger S, Norat T, Riboli E (2018). Tobacco Smoking and the Risk of Sudden Cardiac Death: A Systematic Review and Meta-Analysis of Prospective Studies. Eur J Epidemiol.

[29] Ghearer PJ, De Buyzere ML, Taeymans YM, Gillebert TC, Henriques JP, De Backer G (2006). Risk Factors for Primary Ventricular Fibrillation during Acute Myocardial Infarction: A Systematic Review and Meta-Analysis. Eur Heart J.

[30] Goldenberg I, Moss AJ, McNitt S, Zareba W, Daubert JP, Hall WJ (2006). Cigarette Smoking and the Risk of Supraventricular and Ventricular Tachyarrhythmias in High-Risk Cardiac Patients with Implantable Cardioverter Defibrillators. J Cardiovasc Electrophysiol.

[31] Plank B, Kutyifa V, Moss AJ, Huang DT, Ruwald AC, McNitt S (2014). Smoking is Associated with an Increased Risk of First and Recurrent Ventricular Tachyarrhythmias in Ischemic and Nonischemic Patients with Mild Heart Failure: A MADIT-CRT Substudy. Heart Rhythm.

[32] D'Alessandro A, Boeckelmann I, Hammwöhner M, Goette A (2012). Nicotine, Cigarette Smoking and Cardiac Arrhythmia: An Overview. Eur J Prev Cardiol.

[33] Kang SH, Heo J, Oh IY, Kim J, Lim WH, Cho Y (2016). Ambient Air Pollution and Out-of-Hospital Cardiac Arrest. Int J Cardiol.

[34] Raza A, Bellander T, Bero-Bedada G, Dahlquist M, Hollenberg J, Jonsson M (2014). Short-Term Effects of Air Pollution on Out-of-Hospital Cardiac Arrest in Stockholm. Eur Heart J.

[35] Gentile FR, Primi R, Baldi E, Compagnoni S, Mare C, Contri E (2021). Out-of-Hospital Cardiac Arrest and Ambient Air Pollution: A Dose-Effect Relationship and an Association with OHCA Incidence. PLoS One.

[36] Zhao R, Chen S, Wang W, Huang J, Wang K, Liu L (2017). The Impact of Short-Term Exposure to Air Pollutants on the Onset of Out-of-Hospital Cardiac Arrest: A Systematic Review and Meta-Analysis. Int J Cardiol.

[37] Sielski J, Kazirod-Wolski K, Józwiak MA, Józwiak M (2021). The Influence of Air Pollution by PM2.5, PM10 and Associated Heavy Metals on the Parameters of Out-of-Hospital Cardiac Arrest. Sci Total Environ.

[38] Mehra R (2007). Global public health problem of sudden cardiac death. J Electrocardiol.
